# Autoradiography and preclinical PET studies with radiolabeled asyn-44 and ACI-12589 for imaging α-synuclein

**DOI:** 10.1177/1877718X261431108

**Published:** 2026-04-17

**Authors:** Cassis Varlow, Anna Pees, Jeffrey S Stehouwer, Ann-Kathrin Grotegerd, Daniel Bleher, Dinahlee Saturnino Guarino, Anton Lindberg, Junchao Tong, Brian J Lopresti, Ashley C Knight, Merlin Zabrocki, Arafat Nasser, Vladimir Shalgunov, Elena Ferri, Giuseppe Cannazza, Paul McQuade, Chester A Mathis, Robert H Mach, Umberto M Battisti, Kristina Herfert, Matthias M Herth, Gitte M Knudsen, Neil Vasdev

**Affiliations:** 1 Neurobiology Research Unit, Copenhagen University Hospital Rigshospitalet, Copenhagen, Denmark; 2 Azrieli Centre for Neuro-Radiochemistry, Brain Health Imaging Centre, Campbell Family Mental Health Research Institute, Centre for Addiction and Mental Health (CAMH), Toronto, ON, Canada; 3 Department of Radiology, University of Pittsburgh, Pittsburgh, PA, USA; 4 Werner Siemens Imaging Center, Department of Preclinical Imaging and Radiopharmacy, University of Tuebingen, Tuebingen, Germany; 5 Department of Radiology, Perelman School of Medicine, University of Pennsylvania, Philadelphia, PA, USA; 6 Preclinical and Translational Sciences - Imaging, Takeda Pharmaceutical Company, Cambridge, MA, USA; 7 Department of Drug Design and Pharmacology, Faculty of Health and Medical Sciences, University of Copenhagen, Copenhagen, Denmark; 8 Department of Clinical Physiology, Nuclear Medicine & PET, Copenhagen University Hospital Rigshospitalet, Copenhagen, Denmark; 9 Health Innovative Products and Technologies (HIP-TECH) PhD Program, Department of Life Sciences, University of Modena and Reggio Emilia, Modena, Italy; 10 Department of Life Sciences, University of Modena and Reggio Emilia, Modena, Italy; 11 Faculty of Health and Medical Sciences, University of Copenhagen, Copenhagen, Denmark; 12 Department of Psychiatry, University of Toronto, Toronto, Ontario, Canada

**Keywords:** α-Synuclein, positron emission tomography, asyn-44, Parkinson's disease, multiple system atrophy

## Abstract

**Background:**

Parkinson's disease (PD) and multiple system atrophy (MSA) are considered α-synucleinopathies, characterized by the presence of pathological α-synuclein (α-syn) aggregates. A positron emission tomography (PET) tracer for imaging α-syn aggregates *in vivo* is highly sought after, as disease progression correlates with the accumulation of aggregated α-syn. We recently reported [^18^F]asyn-44 as a radiotracer for α-syn, worthy of evaluation in higher species, based on *in vitro* binding data from human brain tissues and *in vivo* PET imaging studies in rodents.

**Objective:**

[^3^H]ACI-12589 is a promising α-syn PET tracer which recently showed binding in MSA patients but appears to have limited utility in other α-synucleinopathies. Objective 1) compare the *in vitro* binding properties of our lead, [^3^H]asyn-44, to [^3^H]ACI-12589; Objective 2) evaluate [^18^F]asyn-44 and [^18^F]ACI-12589 kinetics by *in vivo* PET imaging in normal rodents; Objective 3) assess pharmacokinetic properties and metabolism of [^18^F]asyn-44 in normal pig and non-human primate (NHP).

**Methods:**

*In vitro* autoradiography with [^3^H]asyn-44 and [^3^H]ACI-12589 was performed to compare radiotracer binding in PD, MSA, Alzheimer's disease and healthy control post-mortem brain tissue. Additionally, preclinical PET imaging was performed in rats with [^18^F]ACI-12589 to compare with our previously reported [^18^F]asyn-44 data. Further evaluation of [^18^F]asyn-44 in higher species was carried out by preclinical PET imaging in pig and NHP with metabolite analysis. Liver microsome assays and mass spectrometry were performed to identify the metabolites formed in NHP.

**Results:**

[^3^H]Asyn-44 and [^3^H]ACI-12589 displayed different binding properties in both PD and MSA tissue, suggesting that the tracers target different binding sites and asyn-44 might therefore be more suited for PD imaging. In the pig, [^18^F]asyn-44 readily entered the brain and no brain penetrant metabolites were observed in arterial blood samples. In the NHP, [^18^F]asyn-44 readily entered the brain but was rapidly metabolized. Radiolabeled metabolites of asyn-44 were proposed and will be considered in the design of future derivatives.

**Conclusions:**

Species differences in metabolism of [^18^F]asyn-44 are observed between pig and NHP, and do not support the further translation of [^18^F]asyn-44. Additionally, autoradiography with [^3^H]asyn-44 revealed low signal specificity and high non-displaceable binding. We report evidence for off-target binding of [^3^H]ACI-12589 to amyloid-β plaques. The limitations of both [^3^H]asyn-44 and [^3^H]ACI-12589 reported here support the development of additional derivatives and structural scaffolds of asyn-44 with the potential to improve radiotracer specificity and selectivity towards α-syn.

## Plain language summary

Comparison of two imaging biomarkers for Parkinson's disease.

Parkinson's disease (PD) and multiple system atrophy (MSA) are brain diseases where the toxic protein, α-synuclein, accumulates in the brain. Researchers are searching for an imaging biomarker to visualize α-synuclein in the brains of living patients, which would help with diagnosis and tracking disease progression. The imaging method used here is called PET, where a drug is labeled with a radioactive isotope (called a radiotracer) that can be tracked in the body after it is administered to a patient. We tested two radiotracers for imaging α-synuclein: 1) our lead radiotracer, 2) a promising radiotracer reported by another research group; for comparison in rats, pigs and non-human primates to understand how they are distributed in the brains of living animals and to identify differences in how they are broken down in the body. Additionally, we tested these radiotracers in post-mortem human brain tissues of patients who had PD, MSA, or Alzheimer's disease and compared them to healthy control patients. We identified differences in the metabolism of our radiotracer between pigs and non-human primates, such that it is unfavourably broken down in non-human primates. Our lead radiotracer also lacked specificity for α-synuclein in human post-mortem brain tissue, and the radiotracer for comparison was found to bind to other aggregated proteins than α-synuclein. Overall, we have provided evidence that the radiotracers evaluated here would not provide an accurate representation of α-synuclein in living patients and further candidates that avoid the troublesome radiometabolism of asyn-44 in the brain should be identified.

## Introduction

α-Synucleinopathies such as Parkinson's disease (PD), Lewy body dementia (LBD) and multiple system atrophy (MSA) collectively share aggregated α-synuclein (α-syn) as a protein pathology believed to contribute to disease pathogenesis.^1,2^ α-Syn is a small presynaptic protein of 140 amino acids. In its native, unfolded state, α-syn can be found abundantly throughout the central nervous system (CNS), primarily localized to the presynaptic membrane, where it plays a role in synaptic plasticity and other brain processes.^3,4^ Although it is unclear how initial misfolding events result in the formation of α-syn insoluble aggregates, α-syn mislocalizes in the cytosol where it adopts beta (or β)-sheet fibrillar conformations.^5–7^ The location and conformation of α-syn aggregates differs among the different diseases: In PD and LBD, α-syn aggregates are mainly found in neuronal cell bodies (Lewy bodies) as well as axons and dendrites (Lewy neurites). In MSA, the α-syn aggregates present as filamentous inclusions in oligodendrocytes (glia cell inclusions; GCI).^8,9^ Distribution and spreading of α-syn follows distinct patterns for PD, LBD and MSA, as for example in the six stages defined by Braak et al. for PD.^10,11^ While spreading, aggregated α-syn acts as a prion-like protein, seeding further aggregation of native α-syn.^12–14^

The distribution pattern of aggregated α-syn provides information on the type of α-synucleinopathy and the disease stage. Therefore, a positron emission tomography (PET) radiotracer that could serve as a biomarker of aggregated α-syn is highly sought after. Despite efforts towards the development of an α-syn PET tracer,^4,5,15,16^ no tracer candidate has yet been identified with clinical potential. Major challenges that have hampered the development of an α-syn PET tracer include the shared β-sheet formation seen with other protein aggregates (amyloid-beta (Aβ), tau) and the low density of α-syn aggregates.^4^ Recently the first α-syn targeted PET tracers were translated to human imaging, symbolizing a significant milestone.^17–19^ For example, Smith et al. reported the development of [^18^F]ACI-12589, which showed promise for the imaging of α-syn in MSA patients but had limited binding in PD, possibly due to the higher α-syn target density in MSA compared to PD.^17^ Endo et al. showed in a preliminary study that their α-syn PET tracer, [^18^F]C05-05, had higher uptake in the midbrain of PD and LBD patients compared to healthy controls,^18^ and Saw et al. found that anle138b derivative [^11^C]MODAG-005 demonstrated binding in a MSA patient.^19^ Additional efforts presented at conferences but not yet reported in peer-reviewed literature include [^11^C]MK-7337 (AD/PD 2025, AAIC 2025, ACS Fall 2025),^20^ [^11^C]SY08 (in a Phase I clinical trial),^21^ and [^18^F]FD4 (in Phase 0 clinical trial).^22^ A recent review has provided a comprehensive overview of the scope of α-synuclein PET tracer development to date.^23^

Our laboratories recently reported another promising α-syn PET tracer, [^18^F]asyn-44.^24^ The *in vitro* evaluation revealed high affinity of the ligand in PD tissue, with high selectivity over Aβ and tau. Specificity of the pathological α-syn signal was confirmed by co-localization with immunohistochemistry and blocking with unlabeled asyn-44. *In vivo* imaging in healthy rodents further supported the suitability of [^18^F]asyn-44 as an α-syn PET tracer with good brain permeability and moderate brain clearance. A concern with [^18^F]asyn-44 in our initial study was the brain penetration of radiometabolites, as *ex vivo* studies revealed >30% radiometabolites (polar and lipophilic) in rodent brain homogenates after 30 min. However, variations in the metabolism of PET tracers among different species are common, and brain-penetrating radiometabolites might not persist in higher species.^25^

The goal of the present work is therefore to further explore the potential of [^18^F]asyn-44 as a putative α-syn radiotracer by: 1) characterizing [^3^H]asyn-44 binding to α-syn in PD, LBD and MSA patient brain tissue using *in vitro* autoradiography; 2) compare the *in vitro* binding of [^3^H]asyn-44 and [^3^H]ACI-12589 in human brain tissues; 3) compare the *in vivo* binding of [^18^F]asyn-44 and [^18^F]ACI-12589 in preclinical PET imaging studies in rodents; 4) assess [^18^F]asyn-44 pharmacokinetics and metabolism using PET imaging in normal pig and non-human primate; and 5) characterize species differences in the metabolic stability of [^18^F]asyn-44 using a liver microsome assay where mass spectrometry was performed on major metabolites in attempt to identify their chemical structures.

## Methods

### General

Authentic standard compounds and precursors for radiolabeling were obtained from MedChem Imaging, Inc. (Boston, USA). The radiolabeled compound [^3^H]asyn-44 (molar activity (A_m_) = 2.146 MBq/µmol) and [^3^H]ACI-12589 (A_m_ = 3.034 MBq/µmol) were custom synthesized by Novandi Chemistry AB (Södertälje, Sweden).

### Human brain samples (University of Pennsylvania)

Post-mortem formalin fixed paraffin embedded (FFPE) tissue sections from different brain regions of donors with confirmed α-synuclein pathology were acquired from the Center for Neurodegenerative Disease Research (CNDR) at the University of Pennsylvania Brain Bank. A neuropathological diagnosis was obtained after review by a board-certified neuropathologist according to consensus criteria.^26^ Each region was assigned a semiquantitative score, i.e., none (0), rare (0.5), mild (1), moderate (2), or severe (3+) for individual lesions based on immunohistochemistry against tau (mouse antibody PHF1, a gift from Dr Peter Davies), Aβ (mouse antibody NAB228, generated in CNDR), a-syn (mouse antibody Syn303, generated at the CNDR) and TDP-43 (rat antibody 1D3, a gift from Elisabeth Kremmer and Dr Manuela Neumann). Patient demographic data are presented in Supplemental Table 1.

### Human brain samples (Germany)

Post-mortem fresh-frozen cryogenic human MSA, AD and HC brain tissue samples were provided as 10 µm sections by the Neurobiobank Muenchen (NBM, Munich, Germany), where cases were collected on the basis of written informed consent according to the guidelines of the ethics committee of the Ludwig-Maximilians-University Munich, Germany (#345-13).

### Human brain samples (Takeda)

Post-mortem fresh-frozen human LBD brain tissue samples were acquired from the Netherlands Brain Bank (NBB, Amsterdam, The Netherlands).

### Animals

*Rats.* Rodent experiments were performed in accordance with animal protocols approved by the Animal Care and Use Committee at the Centre for Addiction and Mental Health (CAMH) with good animal practice (GAP) certification from the Canadian Council on Animal Care (CCAC) and Animals for Research Act registration under the auspices of the Ontario Ministry of Agriculture, Food, and Rural Affairs (OMAFRA).

*Pig.* All animal procedures were performed in accordance with the European Commission's Directive 2010/63/EU, approved by the Danish Council of Animal Ethics (Journal no. 2022-15-0201-01156), and in compliance with the ARRIVE guidelines.

*NHP.* The University of Pittsburgh is fully accredited by the American Association for Accreditation of Laboratory Animal Care (AAALAC), and the animal husbandry staff at the University of Pittsburgh's Division of Laboratory Animal Resources are devoted to fulltime care of animals housed in institutional facilities. Additional information on the veterinary care, housing and enrichment and anesthesia is detailed in the Supplemental Information.

### *In vitro* real-time autoradiography experiments (Penn)

A comparative autoradiography study of [^3^H]asyn-44 and [^3^H]ACI-12589 was carried out in FFPE tissue sections of one PD (cingulate gyrus, PD1) patient and one MSA (dentate gyrus; MSA1) patient. 6-μm-thick deparaffinized sections derived from MSA1 and PD1 brains were first equilibrated for 30 min in 1× PBS (Dulbecco's phosphate buffered saline) and then incubated for 2 h at room temperature with 20 nM [^3^H]asyn-44 or with 20 nM [^3^H]ACI-12589. The sections were rinsed three times in cold buffer 1× PBS + 20% ethanol for 5 min, followed by a quick dip in cold distillated water. Ethanol was included in the wash buffer to reduce non-specific binding.

Slides were then allowed to air-dry before being exposed and scanned in a real-time autoradiography system (BeaQuant instrument, ai4R) for 5 h. Regions of interest (ROIs) were placed over the whole tissue section and quantification of signal was performed by using the image analysis software Beamage (ai4R). Specific binding was determined by subtracting the non-specific signal (NSB) from the total signal and expressed as counts/min/mm^2^. Three adjacent sections from MSA1 and PD1 were used for competition autoradiography studies with unlabeled asyn-44 (500 nM) or ACI-12589 (10 μM). Competitive binding assays were carried out under the same conditions described above.

### *In vitro* autoradiography (Takeda)

*In vitro* autoradiography was performed using 10-μm-thick fresh-frozen sections derived from LBD brains. Brain sections were first equilibrated for 30 min in 1× PBS (Dulbecco's phosphate buffered saline) and then incubated for 2 h at room temperature with 20 nM [^3^H]asyn-44 or with 20 nM [^3^H]ACI-12589. The sections were rinsed three times in cold buffer 1× PBS + 20% ethanol for 5 min, followed by a quick dip in cold distillated water. Non-specific binding was determined using 500 nM unlabeled asyn-44 or 10 µM unlabeled ACI-12589. Slides were then allowed to air-dry before being exposed to a phosphor imaging plate (BAS-IP TR2025, Fuji) with a tritium standard (ART-123; American Radiolabeled Chemicals, St. Louis, MO, USA) for 20 days and scanned in a phosphor imager (BAS 5000, FUJIFILM Life Science, Stamford, CT, USA). For data quantification, ROIs were drawn over the tritium standard to obtain a standard curve and whole tissue section ROI analysis was performed using MCID 7.0 imaging suite (Interfocus Imaging, Cambridge, UK). Specific binding was calculated by subtracting the non-specific binding from the total binding values. After interpolation from the standard curve, raw microcurie/milligram (µCi/mg) specific binding values were divided by the molar activity of the tracer and converted into fmol/mg. Graphical analysis was performed using GraphPad Prism (Version 10.1.1).

### *In vitro* autoradiography experiments (Germany)

*In vitro* autoradiography was performed on fresh-frozen human MSA, cerebellar control, and AD brain tissue. Sections were thawed and subsequently pre-incubated in buffer (30 mM Tris-HCl, 0.1% BSA, pH 7.4) for 25 min at room temperature. This was followed by tracer incubation for 1 h at room temperature. For total binding, the sections were incubated in 10 nM [^3^H]asyn-44 or [^3^H]ACI-12589, respectively. For non-specific binding, adjacent sections were co-incubated with 10 μM of the respective unlabeled compound. After tracer incubation, sections were washed 3 times in ice-cold buffer, followed by a quick wash in ice cold distillated water. The dried sections were then exposed to a phosphor imaging plate (BAS-IP TR2025, Fuji Imaging Plate, VWR, Denmark) with a tritium standard (ART-123 and ART-123A; American Radiolabeled Chemicals, St. Louis, MO, USA) for seven days and scanned in a phosphor imager (BAS 5000, FUJIFILM Life Science, Stamford, CT, USA).

For data quantification, ImageJ software (US National Institutes of Health, Bethesda, MD, USA) was used. Images were converted to 32-bit and the square math function for increasing the grey value range was applied. ROIs were drawn over the tritium standard to obtain a standard curve. ROIs were placed in the white matter of MSA and cerebellar control tissue and in grey and white matter of AD tissue. Specific binding was calculated by subtracting the non-specific binding from the total binding values. After interpolation from the standard curve, the specific binding values were divided by the molar activity of the tracer and converted into fmol/mg. Graphical analysis was performed using GraphPad Prism (Version 10.1.1).

### Microautoradiography (Germany)

For microautoradiography, tissues were thawed and fixed in 4.5% paraformaldehyde (PFA, SAV Liquid Production GmbH, Flintsbach am Inn, Germany) for ten minutes at room temperature. Pre-incubation and washing steps were as above. Brain sections were incubated for 1 h with 60 nM [^3^H]asyn-44 or [^3^H]ACI-12589. Subsequent procedures were performed in a safe light-illuminated dark room. The slides were covered with a 1:1 mixture of distilled water and Ilford K5 emulsion (Agar Scientific Ltd, Stansted, UK) molten in a 40°C water bath and left to dry in an upright position for two hours at room temperature. After drying, the slides were stored in the dark at 4°C for one to two weeks. For development, the slides were incubated for four minutes in Ilford Phenisol at 20°C, followed by one minute in Ilford Ilfostop and four minutes in Ilford Hypam (prepared according to manufacturer's instruction, Agar Scientific Ltd, Stansted, UK). The slides were then washed in running tap water for ten minutes and finally rinsed in distilled water. Immunohistochemistry was performed on the same section as stated above.

### Immunohistochemistry (Penn)

Immunohistochemistry was performed for phosphorylated α-syn at serine 129 (pS129), as this phosphorylation has been identified as a highly toxic event causing degeneration of dopaminergic neurons associated with Parkinson's disease.^27^ Immunohistochemistry was performed on deparaffinized sections adjacent to those used for autoradiography. Sections were permeabilized with 0.1% Triton™ X-100 for 10 min followed by 3 × 5 min washes in PBS Tween 20 (PBST) buffer. Sections underwent hydrogen peroxide blocking for 15 min and PBS + 10% goat serum + 1% bovine serum albumin (BSA) + 0.1% Tween 20 blocking for 1 h at room temperature. Sections were immunostained using an α-syn (pS129) antibody (P-syn/81A; for details see Supplemental Table 2) used at 1:500 dilution overnight at 4°C. After a series of thorough washes with PBST buffer, the slides were incubated with the secondary antibodies Goat Anti-Mouse IgG H&L (horseradish peroxidase (HRP); for details see Supplemental Table 3) at 1: 10000 for 1 h at room temperature. The sections were washed again with PBST buffer for 3 × 5 min, treated with 3,3′-diaminobenzidine (DAB) substrate for 10 min and counterstained by Meyer's hematoxylin dye. Subsequently, the sections were mounted with Limonene mounting media and coverslips for microscopy. Images were captured with a Leica Aperio slide scanner (RRID: SCR_022420) at 10X magnification.

### Immunohistochemistry (Takeda)

Fresh frozen tissue sections previously cryosectioned to a thickness of 10 μm were acclimated to room temperature, post-fixed with acetone for 20 min (−20°C) then permitted to air dry. Tissue sections were encircled with a wax pen well and rinsed in PBS. Endogenous peroxidases were quenched by incubation with BLOXALL (SP-6000-100; Vector Laboratories, Inc., Newark, California, USA) followed by 3 × 5-min washes in PBS. Blocking was performed by incubation with Dako serum free protein block (X090930-2; Agilent, Santa Clara, California, USA) for 30 min followed by an overnight incubation with a primary antibody targeting human phosho-S129 α-syn (ab51253; Abcam, Cambridge, MA, USA) 1:6000 in DAKO antibody diluent (S080983-2; Agilent, Santa Clara, California, USA). Following 3 × 5 min washes in TBST (TBS containing 0.1% Triton-X 100), tissue sections were treated with the ImmPRESS HRP Goat anti-rabbit polymer detection kit per manufacturer's directions (MP-7451-15; Vector Laboratories, Inc.). Samples were washed 3 × 5 min in TBST then treated with the 3,3′-diaminobenzidine (DAB) substrate kit (SK-4105; Vector Laboratories, Inc.) for approximately 90 s. Tissues were counterstained by exposure to Meyer's Hematoxylin (ab245880; Abcam) for 15 s and washed according to the manufacturer's instructions. Imaging was performed using a Leica Aperio Versa slide scanner (Leica; Deer Park, IL, USA) at 1x and 10x magnification.

### Immunohistochemistry (Germany)

On the same or a subsequent section immunohistochemistry was performed. For this, all sections were fixed with 4.5% PFA, followed by a heat-induced antigen retrieval (10 mM sodium citrate, pH 6.0, 30 min, boiling) or antigen retrieval in undiluted formic acid (10 min, room temperature), for α-syn or Aβ, respectively. Following a 5 min wash in PBS, endogenous peroxidase quenching was performed in TBS containing 0.3% H_2_O_2_ and 10% methanol. Subsequently, sections were washed twice in TBS and once in TBS-X (TBS + 0.1% Triton-X + 1% BSA), for 5 min each. Blocking was performed with TBS + 10% normal goat serum + 0.3% Triton-X, for 1 h at room temperature. Sections were immunostained using an α-syn antibody (1:5000 in TBS-X, pSyn#64; 015-25191, FUJIFILM Wako Chemicals Europe GmbH, Neuss, Germany), or an Aβ antibody (1:6000 in TBS-X; 4G8, 800701, BioLegend, Amsterdam, Netherlands), overnight at 4°C. After a series of thorough washes with TBS-X buffer, the slides were incubated with Agilent Dako EnVision HRP (K400111-2; Agilent Technologies Deutschland GmbH, Waldbronn, Germany) for 30 min at room temperature. The sections were washed again with TBS-X buffer for 2 × 105 min and TBS 1 × 10 min, treated with 3,3′-diaminobenzidine (DAB; Pierce™ DAB Substrate Kit) substrate for 10 min and cerebellar sections were counterstained by Meyer's hematoxylin dye. Stained tissues were mounted with Eukitt quick-hardening mounting medium (Fluka Analytical, Munich, Germany). Imaging was performed with a NanoZoomer 2.0 HT (Hamamatsu Photonics K.K., Hamamatsu, Japan) or Zeiss AxioScan digital Slide Scanner Carl Zeiss Microscopy GmbH, Oberkochen, Germany) at 40 × magnification.

### PET/CT acquisition with [^18^F]ACI-12589 in rats and image analysis

[^18^F]ACI-12589 was synthesized according to previously published methods with minor modifications as described in the Supplemental Information with semi-preparative (Supplemental Figure 1) and analytical (Supplemental Figures 2 and 3) HPLC chromatograms shown.^17^ Two healthy adult rats (Sprague Dawley; female, 423 g; male 635 g) underwent PET imaging following bolus injection of [^18^F]ACI-12589 (22.3 MBq and 26.1 MBq, respectively; A_m_ = 35.7 and 15.7 GBq/µmol, respectively, and injected mass 1.5 and 2.6 nmol/kg, respectively) through a catheter in the lateral tail vein. PET images were acquired using a nanoScan™ PET/computed tomography (PET/CT) scanner (Mediso, Budapest, Hungary) with rats anesthetized under isoflurane in O_2_ (1.5–2%, 1 L/min) throughout the imaging session and monitored closely for body temperature and respiration rate. A scout CT was acquired for PET field of view (FOV) positioning, then a material map CT image was acquired for PET corrections for attenuation and scatter as well as for PET/CT co-registration and co-registration with a stereotactic MR atlas of rat brain (Schwarz et al. 2006) used to define anatomical regions of interest (ROI). Dynamic PET scans were acquired for 120 min immediately after i.v. administration of the tracer. Acquired list mode data were sorted into thirty-nine three-dimensional (3D) (3 × 5 s, 3 × 15 s, 3 × 20 s, 7 × 60 s, 17 × 180 s, and 6 × 600 s) true sinograms (ring difference 84). The 3D sinograms were converted in 2D sinograms using Fourier rebinning with corrections for detector geometry, efficiencies, dead-time, attenuation, and scatter before image reconstruction using 2D filtered back-projection (FBKP) with a Hann filter at a cut-off frequency of 5 mm^−1^. Static images corresponding to summation of the complete 120 min emission acquisition as well as those representing the post-injection intervals of 0–2, 2–60 and 60–120 min were reconstructed with the manufacturer's proprietary iterative 3D algorithm (six subsets and four iterations). These images were used for the purposes of PET and CT co-registration, for co-registration of subject's CT with the MR template, and for visualization. All image data were decay-corrected to the time of radiotracer injection. Image analyses and extraction of brain time-activity curves (TACs) from the dynamic FBKP images were performed using VivoQuant^®^ 2020 (2020patch1, Invicro, Needham, MA, USA). Standardized uptake values (SUV) were calculated by normalizing the measured regional radioactivity concentrations by the ratio of the injected radioactivity to the body mass of the animal.

### PET/CT acquisition with [^18^F]asyn-44 in pigs

PET scans were obtained in list-mode with a high-resolution research tomography (HRRT) scanner, with the pig in the prone position. [^18^F]Asyn-44 was given as an intravenous bolus injection (315 MBq; A_m_ = 26.5 GBq/µmol, *n* = 1) and data acquisition began at the time of injection. The HRRT PET data were reconstructed into 45 dynamic frames (6 × 10 s, 6 × 20 s, 6 × 60 s, 8 × 120 s, 19 × 300 s). Images consisted of 207 planes of 256 × 256 voxels of 1.22 × 1.22 × 1.22 mm. Summed images of all coincidence events in the 120 min scan were reconstructed for each pig and used for co-registration to a standardized magnetic resonance imaging (MRI)-based atlas of the Danish Landrace pig brain, similar to that previously published for the Gottingen minipig,^28^ using the software Register, as previously described.^29^ Each co-registration was verified by visual inspection before extraction of TACs from the volumes of interest (VOIs). The extracted regional radioactive concentration (kBq/mL) was normalized to injected dose (MBq) and corrected for animal weight (kg) to give SUV (g/mL). The TACs were determined for the following VOIs: Neocortex and cortical white matter, cerebellum, hippocampus, striatum (caudate and putamen) and thalamus (medial and lateral thalamus).

### Radiolabeled metabolite studies in pigs

Arterial blood samples were manually drawn at 2.5, 5, 10, 20, 30, 50, 70, 90 and 120 min and the radioactivity in whole blood and plasma was measured in a well counter (Hidex AMG, Hidex Oy) that was cross-calibrated to both the HRRT scanner and autosampler. Radiolabeled parent compound and radiolabeled metabolites were measured in plasma using an UltiMate 3000 HPLC system (Thermo Fisher Scientific) with online radioactivity detection (PosiRam 4, LabLogic), as previously described.^28,29^ Arterial blood samples were centrifuged at 3234 × g for 7 min at 4 °C. Plasma was then precipitated with acetonitrile (1:1, v/v) and centrifuged at 8000 × g for 5 min at 4 °C. The resulting plasma supernatant was filtered through a Titan3 17 mm, 0.45 µm PVDF membrane syringe filter (Thermo Fisher Scientific) prior to injection into the HPLC system. The HPLC was equipped with a Luna C18(2) column (250 × 4.6 mm, 5 µm; Phenomenex). The mobile phase consisted of 60% acetonitrile and 40% 0.1 M ammonium formate, delivered at a flow rate of 1.0 mL/min. Injections were performed with a sample volume of 1.5 mL, and the analysis was run at 25 °C for 15 min. HPLC fractions were collected, and the radioactivity was subsequently measured using a gamma counter (Wizard 2480, PerkinElmer). An average parent compound fraction-time curve was generated and the data were fitted to using a biexponential function. The best-fit function was applied to the individual plasma radioactivity concentration curves from the baseline and intervention scans to yield an individual plasma parent compound input function.

### PET/CT acquisition with [^18^F]asyn-44 in NHP

[^18^F]Asyn-44 was synthesized as previously described with minor modifications as described in the Supplemental Information with HPLC chromatograms shown (Supplemental Figures 4–6). The NHP imaging study in a male 12 kg rhesus macaque (macaca mulatta) was carried out in accordance with the guidelines set forth by the Institutional Animal Care and Use Committee at the University of Pittsburgh. The NHP was anesthetized with isoflurane and dynamic [^18^F]asyn-44 PET imaging of the brain was performed using a Siemens Biograph mCT Flow 64-4 R PET/CT scanner (22.1 mm axial FOV, maximum intrinsic spatial resolution of 4.3 mm FWHM).^30^ Following a low-dose CT scan for attenuation correction of PET emission data, [^18^F]asyn-44 was injected intravenously and emission data were acquired in list-mode for a period of 90 min. The injected dose was 105.8 MBq with a molar activity at time of injection of 14.8 GBq/µmol and a radiochemical purity of 99.8%. Emission data were binned into a dynamic series of 32 frames (20 s-10 min) and reconstructed using filtered back projection with Fourier rebinning (FORE + FBP) with standard corrections applied that included attenuation, scatter, electronics dead-time, and physical decay. Reconstructed dynamic PET images were summed into a static image corresponding to the initial ∼20 min of acquisition, which was used to manually define regions-of-interest across several contiguous axial planes for cortex (lateral temporal), cerebellum, and whole brain.

### Radiolabeled metabolite studies in NHP

Venous blood samples (3 mL) were collected at 2, 10, 30, 45, 60, and 90 min post injection. The blood samples were transferred from the sample syringe into centrifuge tubes and centrifuged (Eppendorf MiniSpin 5452) for 45 s at 11,300 × g. The separated plasma (∼0.5 mL) was transferred to a centrifuge tube and diluted with an equal volume of acetonitrile. The resultant solution was vortexed for 1 min and then centrifuged for 45 s at 12,500 rpm. The resulting supernatant was analyzed by reverse-phase HPLC (Phenomenex Luna C18(2), 5 μm, 250 mm × 4.6 mm; 45:55 acetonitrile/0.1 M ammonium bicarbonate, pH 4.6; 2 mL/min) and analyzed using a Raytest 3” × 3” sodium iodide detector with pinhole flow cell (2.0 mL) and Gabi GinaStar software (version 4.07). System suitability of the analytical system was evaluated by injection of an authentic standard of asyn-44 using the same analytical system described above using a Waters UV detector (Model 481, 346 nm).

### Liver microsome assay

Asyn-44 was incubated with cryopreserved rat microsomes using a previously described protocol with marginal changes where necessary.^31^ To summarize, 13 μL of microsome suspension were gently mixed with 5 μL of asyn-44 (50 μM in acetonitrile) in phosphate buffer (432 μL) and 50 μL cofactor (10 mM NADPH) using MTH-100 Thermo Shaker incubator (MIULAB, Hangzhou, Zhejiang, China). Testosterone was used as a positive control and negative controls without the addition of NADPH were incubated for 30 min. Incubation aliquots (100 μL) were quenched with ice-cold acetonitrile containing internal standard (50 μL) and centrifuged for 10 min at 13,000 × *g* with MiniSpin centrifuge (Eppendorf SE, Hamburg, Germany). All reactions were conducted under physiological conditions (37 °C, pH 7.4). Following centrifugation, the supernatant was analysed by LC-HRMS to identify metabolites. Metabolite profiling of asyn-44 was performed using Compound Discoverer 3.3 (Thermo Fisher Scientific, Waltham, MA, USA) with the built-in *MetID* workflow. Additional information on metabolite identification is detailed in the Supplemental Information.

## Results and discussion

### Comparison of [^3^H]asyn-44 vs. [^3^H]ACI-12589 *in vitro* autoradiograms

**Figure 1.**
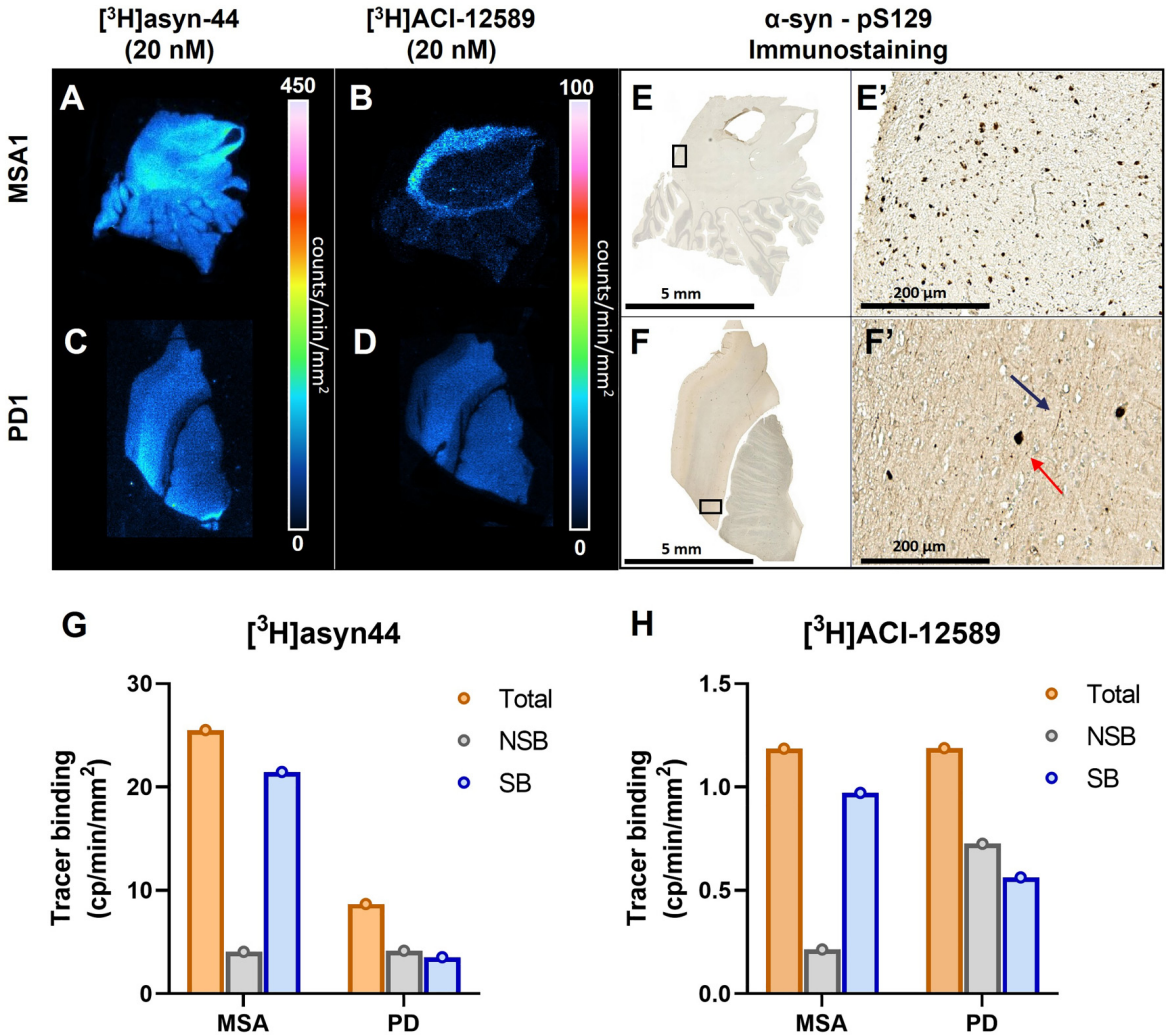
Total binding of [^3^H]asyn-44 and [^3^H]ACI-12589 autoradiography in FFPE postmortem PD and MSA cases. Total (**A**) [^3^H]asyn-44 (20 nM) and (**B**) [^3^H]ACI-12589 (20 nM) binding in MSA1 dentate nucleus. Total (**C**) [^3^H]asyn-44 (20 nM) and (**D**) [^3^H]ACI-12589 (20 nM) binding in PD1 cingulate gyrus. PS129 staining performed on adjacent sections as pathology references in (**E**) MSA1 and (**F**) PD1. Scale bar: 5 mm. A zooming of ROIs is presented in image **E’** and **F’**, showing the presence of GCIs (**E’**) and LBs (**F’**, red arrow) and LNs (**F’**, blue arrow). Scale bar: 200 μm. (**G**) Total binding of [^3^H]asyn-44 (20 nM), non-specific binding in the presence of 500 nM of unlabeled asyn-44 and specific binding expressed as counts/min/mm^2^ in MSA1 and PD1 brains. (**H**) Total binding of [^3^H]ACI-12589 (20 nM), non-specific binding in the presence of 10 µM of unlabeled ACI-12589 and specific binding expressed as counts/min/mm^2^ in MSA1 and PD1 brains. FFPE: formalin fixed paraffin embedded, GCIs: glial cytoplasmic inclusions, LBs: Lewy bodies, MSA: Multiple system atrophy, NSB: non-specific binding, PD: Parkinson's disease, SB: specific binding.

A comparative autoradiography study of [^3^H]asyn-44 and [^3^H]ACI-12589 was carried out in FFPE tissue sections of one PD (cingulate gyrus, PD1) and one MSA (dentate gyrus; MSA1) case. Comparing the autoradiographic distributions of [^3^H]asyn-44 vs. [^3^H]ACI-12589 in PD and MSA tissues, we observed different binding patterns as well as a much higher signal intensity for [^3^H]asyn-44 compared to [^3^H]ACI-12589 assessed visually (Figure 1A-D) as well as semi-quantitatively (Figure 1G,H). In the self-blocking experiments with the respective unlabeled reference standards, [^3^H]asyn-44 demonstrated 84% displacement in MSA tissue and 52% displacement in PD tissue, whereas [^3^H]ACI-12589 showed comparable displacement (80%) in MSA tissue but lower (39%) displacement in PD tissue. When blocking [^3^H]asyn-44 with unlabeled ACI-12589, the signal was reduced by 64% in MSA tissue but non-specific binding increased in PD tissue (Supplemental Figure 7A,C). When blocking [^3^H]ACI-12589 with asyn-44 no displacement was detected in any tissue and heterologous blocking was shown to increase non-specific binding in MSA1. A possible explanation of the increased non-specific binding when performing heterologous blocking may be due to the different binding sites of the radiotracer and the blocking compound, creating the occurrence of a negative cooperativity between the two (Supplemental Figure 7B,D). These data indicate that asyn-44 and ACI-12589 have different binding properties in both in MSA and PD tissues and suggests that these tracers might target or access different binding sites of α-syn aggregates. Immunostaining revealed the presence of positive pS129 α-syn GCIs in the cerebellar white matter of MSA1 (**Figure 1E**). In PD1, the cingulate gyrus is one of the first cortical structures to develop Lewy pathology in PD, and this region is one of the most severely affected.^32^ Immunohistochemistry in the cingulate gyrus of a PD case revealed the presence of LBs and LNs (**Figure 1F**). Both [^3^H]asyn-44 and [^3^H]ACI-12589 displayed positive radiotracer signal in both MSA and PD in regions with α-syn pathology.

**Figure 2.**
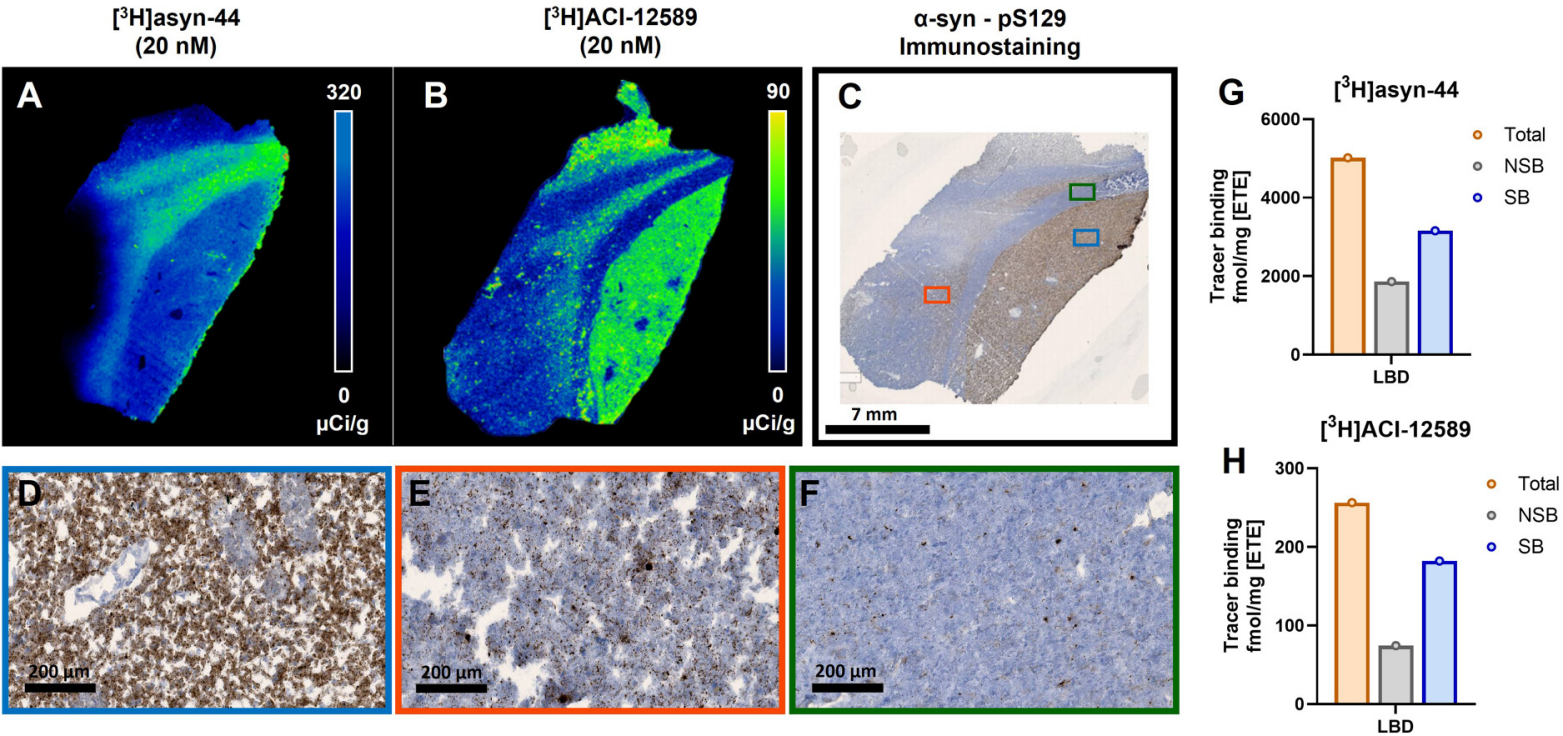
Total binding of [^3^H]asyn-44 and [^3^H]ACI-12589 autoradiography in fresh-frozen postmortem LBD brain. (A) Total [^3^H]asyn-44 (20 nM) and (B) [^3^H]ACI-12589 (20 nM) in LBD1 putamen. Calibration bar shown in µCi/g, note scaling differs between (A) and (B). (C) PSer129 a-syn staining performed on adjacent sections as pathology references. Red, green and blue boxes highlight a-syn pathology throughout the grey and white matter. 7 mm scale bar. (D) Enlarged blue box shows a representative area of dense immunostaining which aligns more closely with autoradiography signal intensity of [^3^H]ACI-12589 but not that of [^3^H]asyn44 (E) Enlarged red box shows moderate IHC density aligning with the less intense, more punctate appearance of [^3^H]ACI-12589 binding, (F) Enlarged green box shows the least dense IHC staining pattern aligning with [^3^H]ACI-12589 intensity and not the binding intensity of [^3^H]asyn44. 200 um scale bar. Quantitation of (G) [^3^H]asyn44 and (H) [^3^H]ACI-12589 binding expressed as fmol/mg ETE in LBD1 brains. α-syn: α-synuclein; LBD: Lewy body dementia; ETE: Estimated tissue equivalent.

[^3^H]Asyn-44 and [^3^H]ACI-12589 autoradiography was also carried out in tissue sections of one fresh frozen LBD case (putamen; LBD1). Consistent with results seen in PD and MSA, different binding patterns were observed for both tracers, as well as a much higher signal intensity for [^3^H]asyn-44 compared to [^3^H]ACI-12589, seen visually (**Figure 2A,B**) and semi-quantitatively (**Figure 2G,H**). By self-blocking with the respective unlabeled reference standards, [^3^H]asyn-44 total binding (5016.52 fmol/mg) was reduced 63%. [^3^H]ACI-12589 demonstrated a comparable specific binding window (71%), however, absolute binding values were much lower, with a specific binding signal of 127.22 fmol/mg in LBD1. Overall, these data continue to indicate differential binding properties. Immunohistochemistry showed positive pS129 α-syn staining, showing the presence of both Lewy bodies and Lewy neurites in the grey matter of the putamen, these areas of dense immunostaining align more closely with the autoradiography signal intensity of [^3^H]ACI-12589 but not that of [^3^H]asyn44 (**Figure 2D,E**). Phosphorylated α-syn-immunopositive inclusions are also seen in glial cells in the white matter, although much less dense than immunostaining in the grey matter, and this pattern aligns with [^3^H]ACI-12589 intensity and not the binding intensity of [^3^H]asyn-44 (**Figure 2F**). Both [^3^H]asyn-44 and [^3^H]ACI-12589 displayed positive radiotracer signal in LBD in regions with α-syn pathology, but overall, [^3^H]ACI-12589 aligns more closely with α-syn pathology compared to [^3^H]asyn-44.

**Figure 3.**
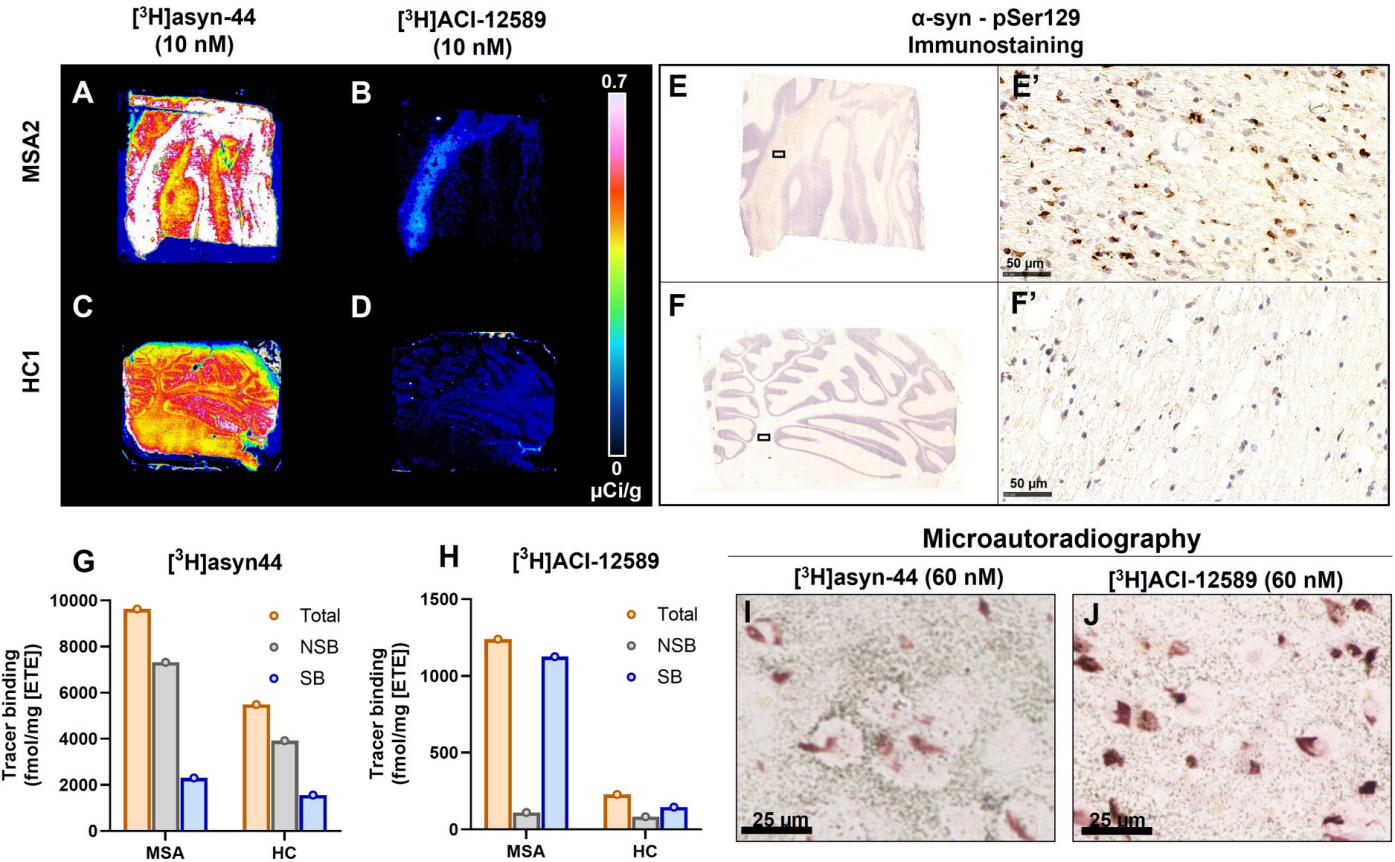
Total binding of [^3^H]asyn44 and [^3^H]ACI-12589 (micro)autoradiography in fresh-frozen postmortem MSA and healthy control cases. Total [^3^H]asyn-44 (10 nM) binding in (A) MSA2 cerebellum and (C) healthy control (HC) cerebellum, respectively. [^3^H]ACI-12589 (10 nM) binding in (B) MSA2 cerebellum and (D) HC1 cerebellum, respectively. Calibration bar shown in µCi/g. PSer129 α-syn staining performed on same or adjacent sections as pathology references in (E) MSA2 and (F) healthy control cerebellum. (E’) The presence of GCIs is shown in MSA2 and (F’) not in HC1. Scale bar: 50 μm. (G) Total binding of [^3^H]asyn-44 (10 nM), non-specific binding in the presence of 500 nM of unlabeled asyn-44 and specific binding expressed as fmol/mg ETE in MSA2 and HC1 brains. (H) Total binding of [^3^H]ACI-12589 (10 nM), non-specific binding in the presence of 10 µM of unlabeled ACI-12589 and specific binding expressed as fmol/mg ETE in MSA2 and HC1 brains. Microautoradiography of (H) [^3^H]asyn-44 (60 nM) and (I) [^3^H]ACI-12589 (60 nM) binding in MSA2 cerebellum. α-syn: α-synuclein HC: healthy control, ETE: Estimated tissue equivalent, GCI: Glia cell inclusions MSA: Multiple system atrophy.

[^3^H]Asyn-44 and [^3^H]ACI-12589 were also compared in tissue sections from one MSA (MSA2; cerebellum) and one healthy control (HC1; cerebellum) case. Again, different binding patterns were observed and [^3^H]asyn-44 displayed much higher signal intensity compared to [^3^H]ACI-12589. This was both assessed visually (**Figure 3A-D**) as well as semi-quantitatively (**Figure 3G,H**). In a self-blocking experiment with the respective unlabeled reference standards, [^3^H]asyn-44 demonstrated a much lower specific binding window when compared to MSA1, detailed above. In the second MSA case (MSA2) [^3^H]asyn-44 specific binding was 2305.70 fmol/mg, but this specific signal was only 24% of the total binding (**Figure 3G**). One possible explanation for this discrepancy could be due to the different tissue regions between MSA1 and MSA2 (cerebellum vs. dentate gyrus, respectively), although both regions have α-syn pathology confirmed by immunostaining. Immunostaining revealed the presence of positive pS129 α-syn GCIs in the cerebellar white matter of MSA2 (**Figure 3E**). Whereas in HC1 tissue, no α-syn was detected by pS129 immunostaining (**Figure 3F**), [^3^H]asyn-44 specific binding was 1566.36 fmol/mg (29% of total signal, **Figure 3G**). This indicates that whether α-syn pathology is present or not, [^3^H]asyn-44 binds to the tissues to the same degree. These data suggest a lack of specificity of [^3^H]asyn-44 binding, which may be attributed to non-displaceable binding at 500 nM of unlabeled asyn-44. [^3^H]ACI-12589 specific binding in MSA2 was 1128.43 fmol/mg (91% of total signal, **Figure 3H**) at 10 μM of unlabeled ACI-12589, a much higher percent specific signal than that observed for [^3^H]asyn-44. In HC tissue, [^3^H]ACI-12589 specific binding was 145.70 fmol/mg (64% of total signal, **Figure 3H**), which is less than that observed in MSA tissue, however still of note for tissue that does not have α-syn pathology. Since the majority of this signal is specific, there may be off-target binding to consider. Microautoradiography was performed with both [^3^H]asyn-44 and [^3^H]ACI-12589 to provide a higher spatial resolution than the macro autoradiography presented thus far. Microautoradiography allows for the localization of radiolabeled tracers to specific subcellular structures and here confirms [^3^H]asyn-44 (**Figure 3I**) and [^3^H]ACI-12589 (**Figure 3J**) binding to α-syn inclusions in MSA cerebellum. Overall, these data reveal both [^3^H]asyn-44 and [^3^H]ACI-12589 suffer from distinct limitations.

**Figure 4.**
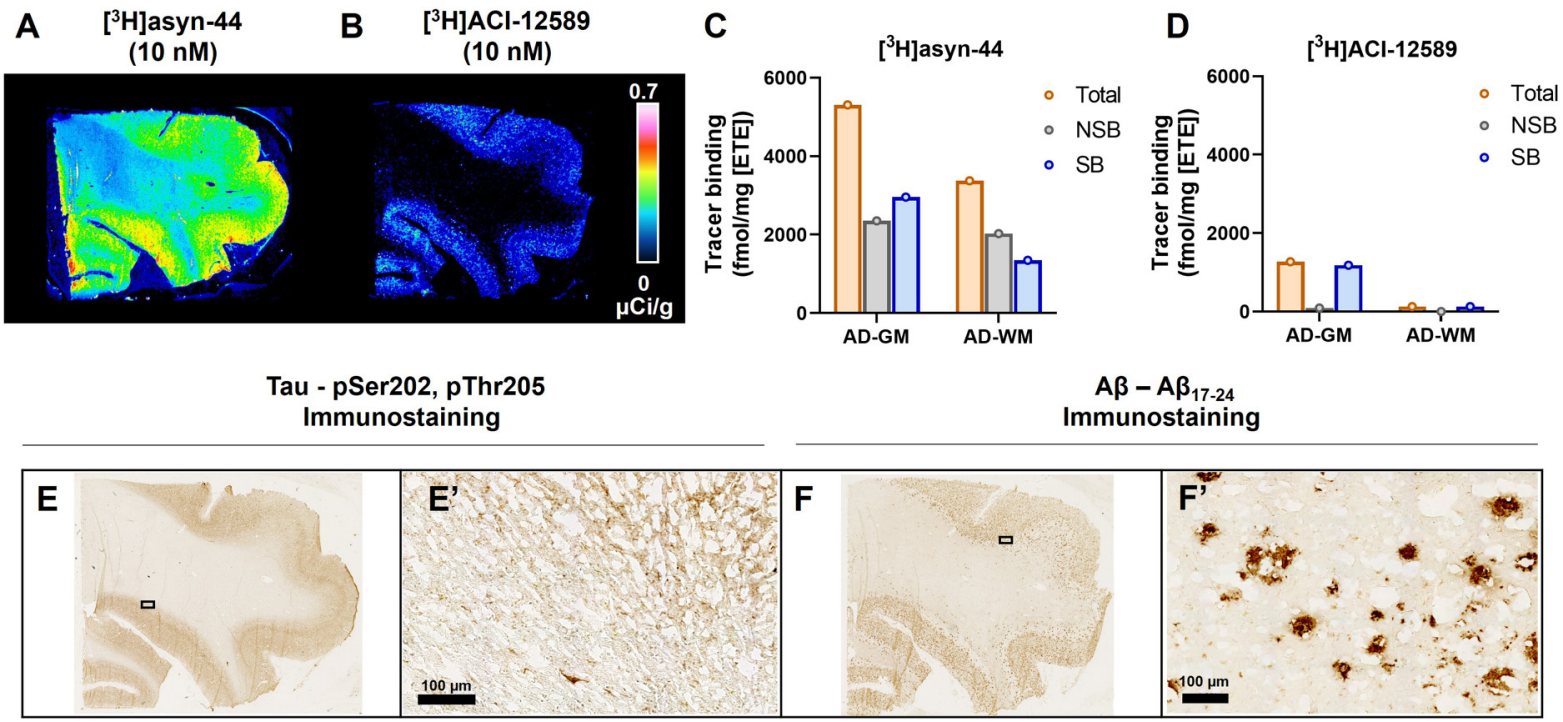
Total binding of [^3^H]asyn-44 and [^3^H]ACI-12589 autoradiography in a fresh-frozen postmortem AD case. Total (A) [^3^H]asyn-44 (10 nM) and (B) [^3^H]ACI-12589 (10 nM) binding in AD1 frontal cortex. Calibration bar in µCi/g. Quantitation of total, non-specific (10 nM hot + 10 µM cold) and specific binding expressed as fmol/mg ETE in AD1 frontal cortex with (C) [^3^H]asyn-44 and (D) [^3^H]ACI-12589. PSer202, pThr205 tau and Aβ_17−24_ staining performed on same or adjacent sections as pathology references in AD1 tissue in (E) and (F), respectively. A zooming of ROIs is presented in image E’ an F’ showing the presence of tau- and Aβ-pathology. Scale bar: 100 μm. Aβ: Amyloid-β, AD: Alzheimer's disease, ETE: Estimated tissue equivalent, GM: Grey matter, WM: White matter.

We evaluated whether there was any binding to other proteinopathies with [^3^H]ACI-12589 and [^3^H]asyn-44 by autoradiography comparison in tissue sections from one Alzheimer's disease case (AD1; frontal cortex). Consistent with MSA, PD and HC, different binding patterns were observed and [^3^H]asyn-44 displayed much higher signal intensity compared to [^3^H]ACI-12589 (**Figure 4A,B**). Semi-quantitation showed [^3^H]asyn-44 bound with 56% specific binding in AD1 grey matter and to a lesser extent (40%) in the white matter (**Figure 4C**). This is in line with the data from HC, MSA and PD, where [^3^H]asyn-44 appears to be limited by non-displaceable binding as seen by over half the signal attributed to non-specific binding. [^3^H]ACI-12589 displayed 93% specific binding in AD1 grey matter and 100% in white matter, although the absolute binding values were 10-times lower in the white matter (**Figure 4D**). Immunostaining was performed to confirm the presence and distribution of tau protein aggregates and amyloid-β plaques (**Figure 4E,F**). In the AD case, the distribution of [^3^H]ACI-12589 closely aligned with the distribution of amyloid-β plaques, further supporting that [^3^H]ACI-12589 binding is influenced by off-target interactions and providing evidence that amyloid-β plaques contribute to this off-target binding.

### Comparison of [^18^F]asyn-44 vs. [^18^F]ACI-12589 by PET imaging in rats

**Figure 5.**
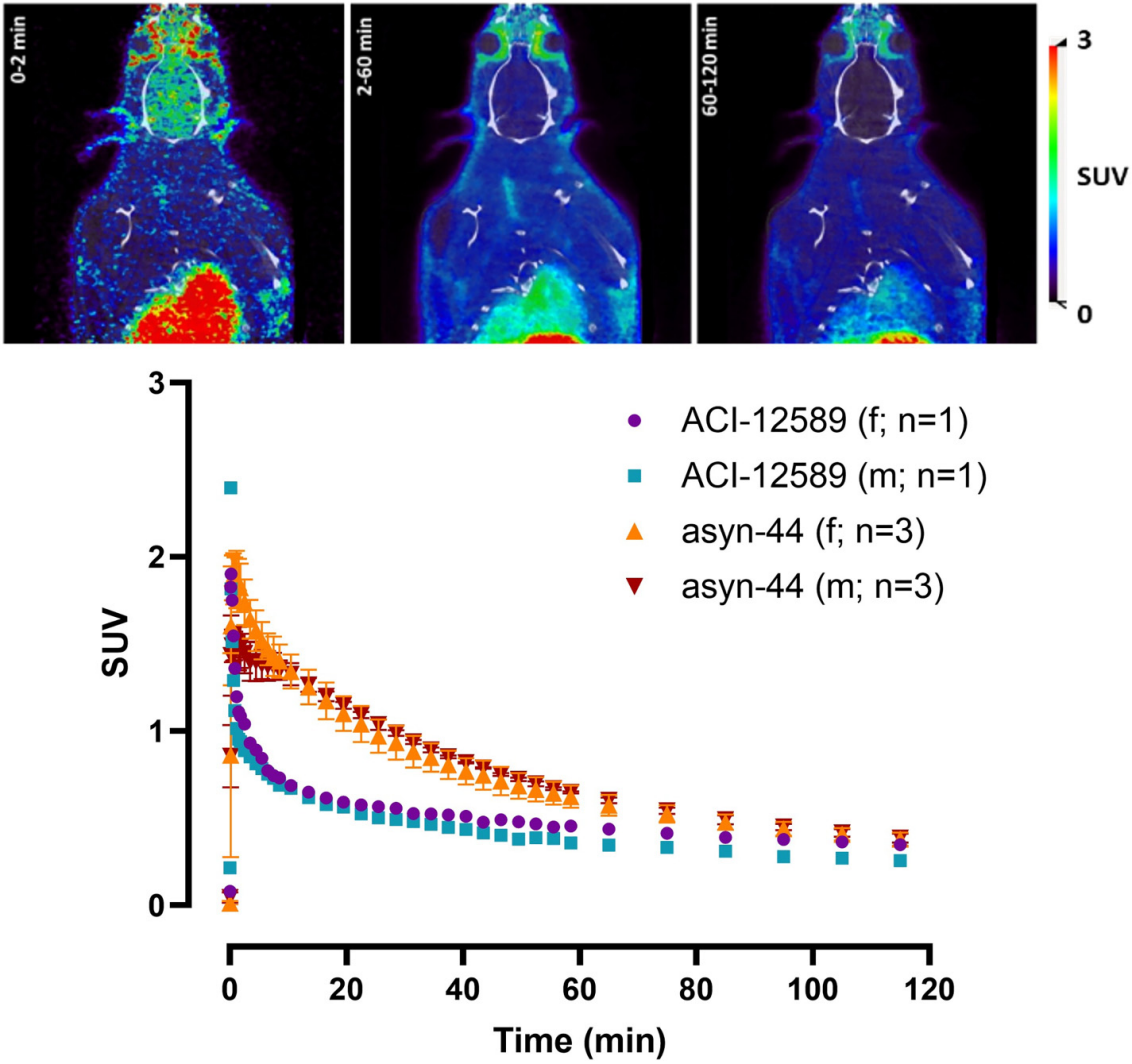
*In vivo* brain PET study of [^18^F]ACI-12589 compared with [^18^F]asyn-44 in rats. Top: Representative example of summed brain images (0–2, 2–60, and 60–120 min) with [^18^F]ACI-12589 (this study); Bottom: Time-activity curves in whole brain of male and female healthy rats with [^18^F]ACI-12589 (this study) and [^18^F]asyn-44, previously reported.^24^ Open access under a Creative Commons Attribution 4.0 International License (CC BY 4.0) (https://creativecommons.org/licenses/by/4.0/). Figure has been adapted.

To evaluate the *in vivo* properties of [^18^F]ACI-12589 in rodents, we carried out a PET imaging study in healthy rats using the same procedure as in our previously reported study with [^18^F]asyn-44.^24^ Two rats (1 x male, 1 x female) were scanned for 2 h after injection of [^18^F]ACI-12589. **Figure 5** shows summed images of [^18^F]ACI-12589 binding in rat brain and TACs of both male and female animals (purple and blue), compared to the previously reported average TACs of [^18^F]asyn-44 (orange and red).^24^ Both tracers had comparable initial uptake of around 2 SUV in the whole brain, but the rate of clearance for [^18^F]ACI-12589 was much more rapid. The differences in the rates of clearance are shown in the TACs (**Figure 5**) and this is also demonstrated by the peak-to-retention ratio, which was 3.9 for [^18^F]ACI-12589 and 1.9 for [^18^F]asyn-44. [^18^F]ACI-12589 SUVs were reduced to below 0.5 after about 30–40 min, [^18^F]asyn-44 reached the same levels after 90 min.

### PET imaging of [^18^F]asyn-44 in pigs

**Figure 6.**
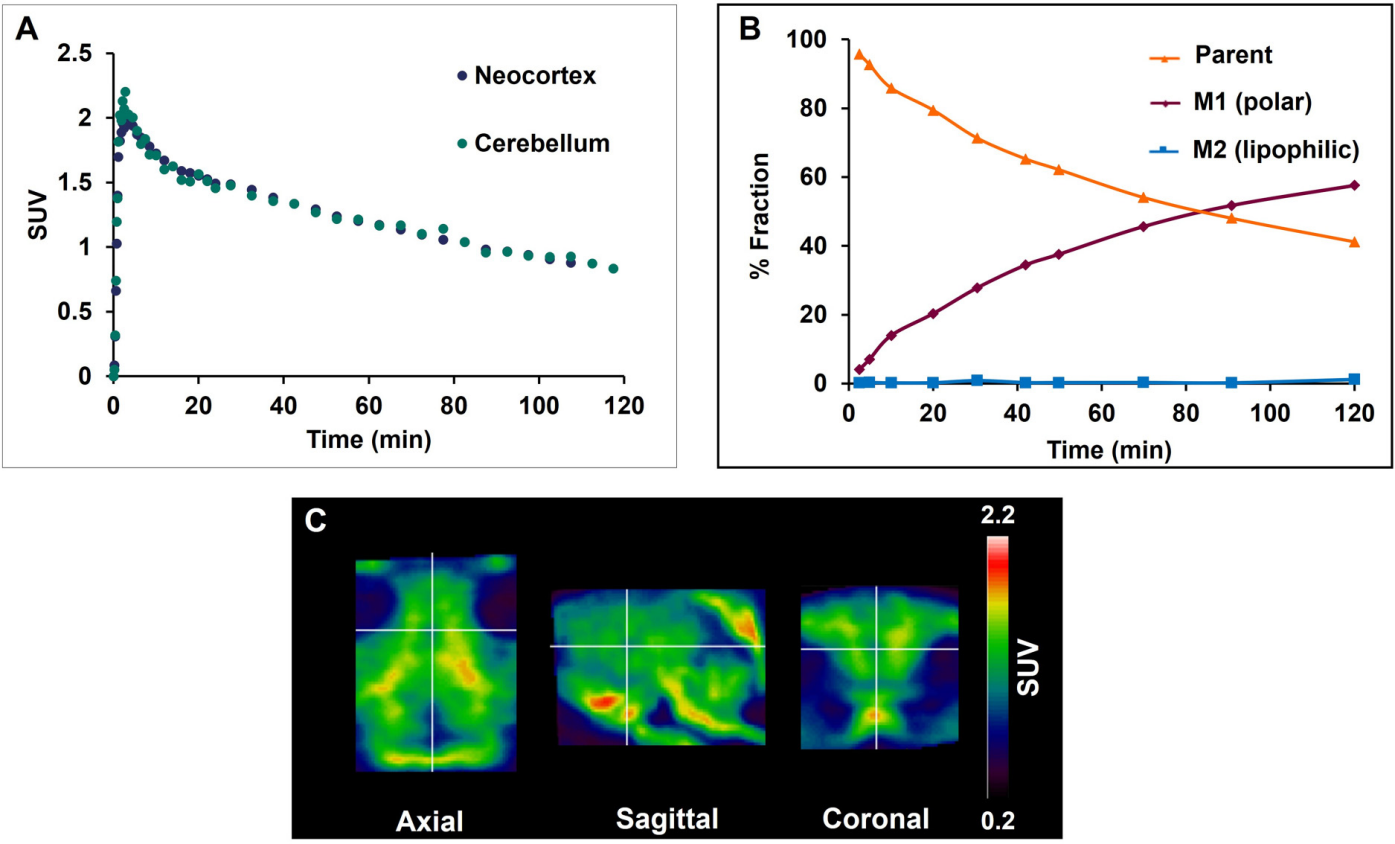
*In vivo* [^18^F]asyn-44 imaging in pig. (A) Regional time-activity curves (TACs) of [^18^F]asyn-44 at baseline (n = 1). (B) Percentage of [^18^F]asyn-44 parent fraction in arterial blood samples over the duration of the 2 h PET scan. The presence of potential metabolites (M1) eluting before [^18^F]asyn-44 and the lack of metabolites eluting later than [^18^F]asyn-44 (M2) over time. (C) Representative axial, sagittal and coronal orientation of 6–90 min summed SUV scaled PET images.

Baseline PET imaging in pigs with [^18^F]asyn-44 displayed moderate brain penetration with a peak standardized uptake value (SUV) of 2.2 in the cerebellum (**Figure 6A**), which was similar to the peak uptake seen in healthy rodents. Clearance of [^18^F]asyn-44 from the brain was relatively similar to that seen in rats, where the TACs show a steeper clearance between the peak and 20 min that becomes less steep after 20 min of scanning, however the TACs continue to steadily decrease. As the PET studies were all carried out in naïve animals, with no α-synuclein pathology, there should be no specific binding of the radiotracer in the brain. For instance, the radiotracer signal in the lateral ventricles is similar to all other brain regions. The percent parent fraction over the duration of the scan shows 48% of the radiotracer is present at 90 min, indicating adequate stability of [^18^F]Asyn-44 in pigs (Figure 5B). Most notably, there was no distinct peak formation as seen in the chromatograms of the metabolite profile (Supplemental Figure 8), however some formation of polar metabolites may contribute to the noise seen on the chromatograms at ∼30 min-onward, eluting before the [^18^F]asyn-44 peak. Summed images of the baseline scan show widespread distribution (**Figure 6C**).

Although we cannot exclude that the noise seen on the chromatogram could potentially be attributed to the formation of polar metabolites, seen on the chromatograms between the solvent front and [^18^F]asyn-44 eluting at later timepoints, there are no distinct peaks corresponding to any lipophilic (or polar) metabolites. This is contradictory to what was observed in rodents. One additional explanation for the noise observed could be due to injection of high sample volume onto the HPLC to ensure enough activity for detection. High volume will in particularly cause diffusion and elution of more polar compounds showing this smeared-like band (Supplemental Figure 8). Radioactivity in the plasma fraction after precipitation was 94%. Hence, the majority of the activity is in the supernatant. The final 6% could be [^18^F]asyn-44 in the cell/protein fraction and/or [^18^F]asyn-44 from residual supernatant that was not completely removed. The plasma free fraction was max. 1.2% after 2 h, however, at this time it had not reached equilibrium.

### PET imaging of [^18^F]asyn-44 in NHP

**Figure 7.**
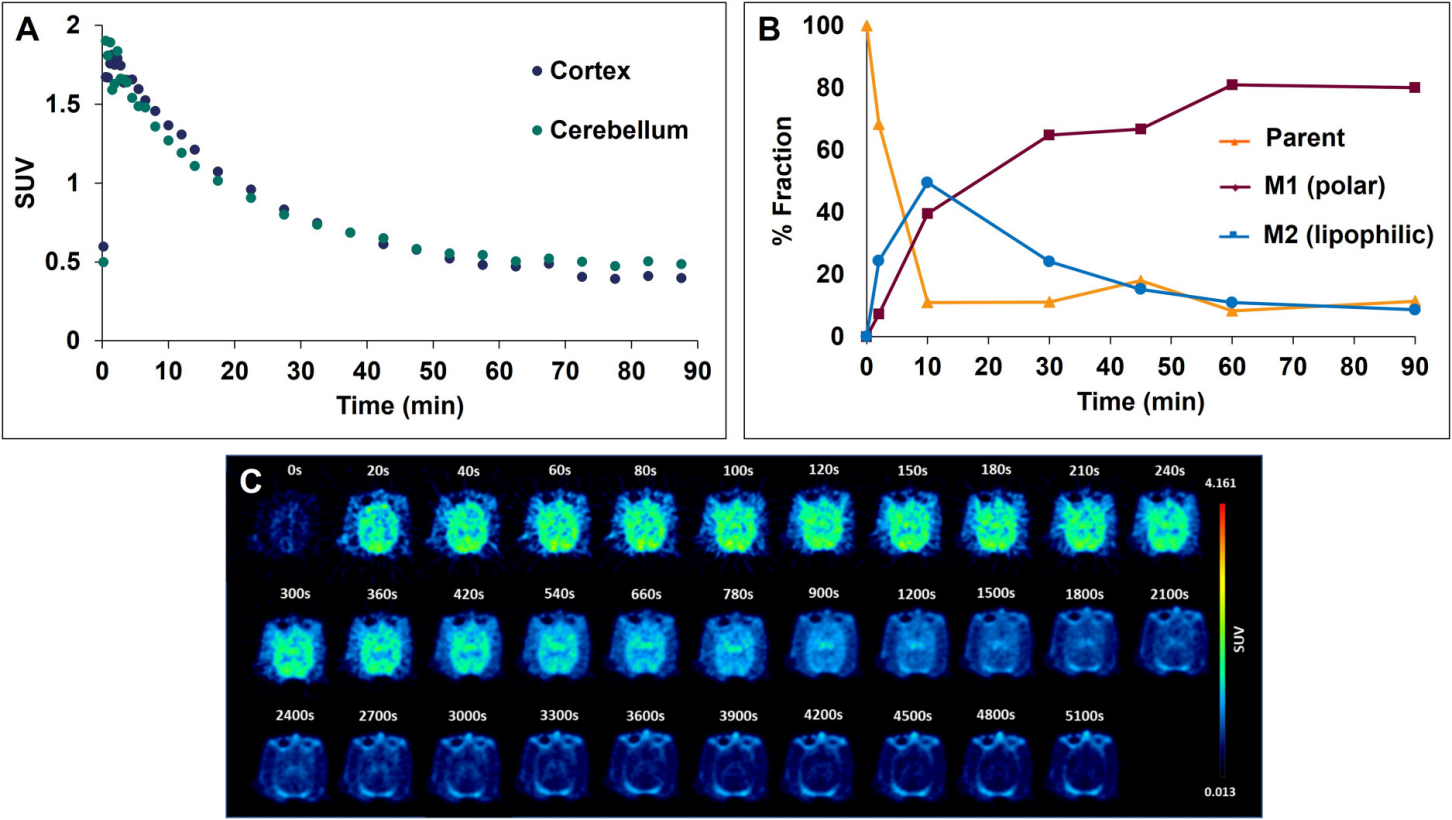
*In vivo* brain PET study of [^18^F]asyn-44 in NHPs. (A) Time-activity curves of [^18^F]asyn-44 in whole brain, cortex and cerebellum; (B) Fraction of parent compound ([^18^F]asyn-44) in venous blood depending on time post injection; (C) Time course of axial images of NHP brain.

Dynamic PET imaging with [^18^F]asyn-44 was conducted in a rhesus macaque using a Siemens Biograph mCT over 90 min. The injected dose was 105.8 MBq with an apparent molar activity of 14.8 GBq/µmol at time of injection. The tracer displayed good brain uptake with peak SUVs of >1.5 within the first 5 min, followed by more rapid clearance than what was observed in pigs (**Figure 7A,C**). The TACs were flattening out beyond 60 min. Metabolite analysis of venous blood samples revealed that [^18^F]asyn-44 was quickly metabolized into polar and a lipophilic metabolite (**Figure 7B;** Supplemental Figure 9). The lipophilic metabolite was more predominant at early timepoints (49.5% at 10 min), whereas the polar metabolite(s) were the almost exclusive species at later time points (80.1% at 90 min). This indicates that [^18^F]asyn-44 might be first metabolized to the lipophilic metabolite, which is then subsequently metabolized into the polar ones. At the end of the scan, only 11.4% intact [^18^F]asyn-44 was still left in the blood. The lipophilic metabolite might be able to cross the blood-brain barrier, which could be a potential reason for the flattening of the TACs after 60 min. An additional finding was the presence of bone uptake at late scan time points (>70 min), indicating some defluorination of the tracer. This is an interesting finding because defluorination has not been observed in rodents during the 120 min scan interval. Overall, the potential brain-penetrant metabolite and defluorination suggest that [^18^F]asyn-44 is not a very promising candidate for human imaging studies. While the scaffold seems to have good brain-penetrating properties, further derivatization towards a molecule with improved metabolic stability is needed.

### Metabolite identification

**Figure 8.**
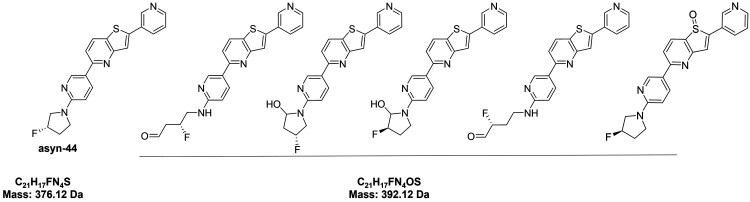
Potential Asyn-44 metabolites. Chemical structure of asyn-44 and most probable oxidation products.

To identify metabolites of asyn-44, chromatograms from rat liver microsomal incubations at 0 and 30 min were processed and based on chromatographic peak area, the most abundant metabolite of asyn-44 (M1; Supplemental Figure 1 **1A**) eluted at a retention time (*R_T_*) of 7.61 min (Supplemental Figure 1 **1B**). It was detected as the protonated molecular ion [M + H]^+^ at *m/z* 393.1173, corresponding to an oxidized species exhibiting a + 16 Da mass shift relative to the parent compound. MS/MS analysis of the precursor ion yielded a characteristic fragmentation pattern (Supplemental Figure 11C), consistent with structure M1 of the *in silico*–predicted metabolite (Supplemental Figure 11A and Supplemental Table 4). The Figure shows the fragmentation pattern with the proposed structures for the major fragments annotated in the spectrum (Supplemental Figure 11C). Characterization of two additional potential metabolites are detailed in the Supplemental (Supplemental Figure 11D-H). Notably, the major oxidized metabolite, characterized by a *R_T_* = 7.61 min, eluted prior to the parent compound, indicating increased hydrophilicity. In addition, a minor oxidized species was detected with a *R_T_* = 8.75 min, eluting shortly after asyn-44, representing greater lipophilicity. Therefore, two oxidation products are likely: one more hydrophilic and the other more lipophilic. This is consistent with the metabolite profile observed in rat brain homogenates and NHP venous samples. The probable oxidation sites were identified and potential structures of these metabolites are shown in **Figure 8**. With likely metabolites identified, future studies will focus on radiolabeling the most probably metabolite structures to assess brain penetration in higher species and determine whether these radiometabolites are definitively a confounding factor of [^18^F]asyn-44 imaging analysis. Additionally, identification of sites susceptible to oxidation will guide the design and selection of future derivatives for evaluation as potential α-synuclein PET tracer candidates to avoid such troublesome brain penetrating radiometabolites.^33^

## Conclusion

[^18^F]Asyn-44 is a promising scaffold for binding to α-syn, but limitations such as poor *in vivo* stability in non-human primate PET imaging studies, including some defluorination at later timepoints, and high non-displaceable binding seen in MSA, PD, HC and AD tissues by autoradiography, preclude translation to humans. [^3^H]Asyn-44 specific binding was highly variable between MSA cases and a larger sample size would be needed to determine the utility of this radiotracer as an *in vitro* screening tool. Additionally, [^3^H]ACI-12589 binding by autoradiography aligned more closely with α-syn pathology, compared to [^3^H]asyn-44. Future work will focus on developing stable derivatives of asyn-44 for PET.

## Supplemental Material

sj-docx-1-pkn-10.1177_1877718X261431108 - Supplemental material for Autoradiography and preclinical PET studies with radiolabeled asyn-44 and ACI-12589 for imaging α-synuclein Supplemental material, sj-docx-1-pkn-10.1177_1877718X261431108 for Autoradiography and preclinical PET studies with radiolabeled asyn-44 and ACI-12589 for imaging α-synuclein by Cassis Varlow, Anna Pees, Jeffrey S Stehouwer, Ann-Kathrin Grotegerd, Daniel Bleher, Dinahlee Saturnino Guarino, Anton Lindberg, Junchao Tong, Brian J Lopresti, Ashley C Knight, Merlin Zabrocki, Arafat Nasser, Vladimir Shalgunov, Elena Ferri, Giuseppe Cannazza, Paul McQuade, Chester A Mathis, Robert H Mach, Umberto M Battisti, Kristina Herfert, Matthias M Herth, Gitte M Knudsen, and Neil Vasdev in Journal of Parkinson's Disease
