# Supplementary material for: Autoradiography and preclinical PET studies with radiolabeled asyn-44 and ACI-12589 for imaging α-synuclein
Source: J Parkinsons Dis. 2026 Apr 17:1877718X261431108. Online ahead of print. doi: 10.1177/1877718X261431108 (PMC13224086; doi:10.1177/1877718X261431108)
Supplement: Supplementary file 1 [file sj-docx-1-pkn-10.1177_1877718X261431108.docx]

*Supporting information*

*for*

**Autoradiography and Preclinical PET Studies with Radiolabeled Asyn-44 and ACI-12589 for Imaging α-Synuclein**

Cassis Varlow,^1^ Anna Pees,^2^ Jeffrey S. Stehouwer,^3^ Ann-Kathrin Grotegerd,^4^ Daniel Bleher,^4^ Dinahlee Saturnino Guarino,^5^ Anton Lindberg,^2^ Junchao Tong,^2^ Brian J. Lopresti,^3^ Ashley C. Knight,^6^ Merlin Zabrocki,^7^ Arafat Nasser,^1^ Vladimir Shalgunov,^8^ Elena Ferri^9,10^, Giuseppe Cannazza,^10^ Paul McQuade,^6^ Chester A. Mathis,^3^ Robert H. Mach,^5^ Umberto M. Battisti,^7^ Kristina Herfert,^4^ Matthias M. Herth,^7^ Gitte M. Knudsen,^1,11^ and Neil Vasdev^2,12^*

^1^Neurobiology Research Unit, Copenhagen University Hospital Rigshospitalet, Copenhagen, Denmark

^2^Azrieli Centre for Neuro-Radiochemistry, Brain Health Imaging Centre, Campbell Family Mental Health Research Institute, Centre for Addiction and Mental Health (CAMH), Toronto, ON, Canada

^3^Department of Radiology, University of Pittsburgh, Pittsburgh, PA, United States

^4^Department of Preclinical Imaging and Radiopharmacy, Werner Siemens Imaging Center, Eberhard Karls University Tuebingen, Tuebingen, Germany

^5^Department of Radiology, Perelman School of Medicine, University of Pennsylvania, Philadelphia, PA, USA

^6^Takeda Pharmaceutical Company, Cambridge, MA, USA

^7^Department of Drug Design and Pharmacology, Faculty of Health and Medical Sciences, University of Copenhagen, Copenhagen, Denmark

^8^Department of Clinical Physiology, Nuclear Medicine & PET, Copenhagen University Hospital Rigshospitalet, Copenhagen, Denmark

^9^Health Innovative Products and Technologies (HIP-TECH) PhD Program, Department of Life Sciences, University of Modena and Reggio Emilia, Modena, Italy

^10^Department of Life Sciences, University of Modena and Reggio Emilia, Modena, Italy

^11^Faculty of Health and Medical Sciences, University of Copenhagen, Copenhagen, Denmark

^12^Department of Psychiatry, University of Toronto, Ontario, Canada

Contents

[Materials and Methods	3](#_Toc218676617)

[General	3](#_Toc218676618)

[Rats	3](#_Toc218676619)

[Pig	3](#_Toc218676620)

[NHP veterinary care, housing, and enrichment program	3](#_Toc218676621)

[[^18^F]Asyn-44 radiosynthesis for rat imaging (CAMH)	4](#_Toc218676622)

[[^18^F]ACI-12589 radiosynthesis (CAMH)	4](#_Toc218676623)

[[^18^F]Asyn-44 radiosynthesis for pig imaging (Copenhagen University Hospital)	4](#_Toc218676624)

[[^18^F]Asyn-44 radiosynthesis for NHP imaging (University of Pittsburgh)	5](#_Toc218676625)

[Radiometabolite Identification	5](#_Toc218676626)

[Brain Tissue Patient Demographics	7](#_Toc218676627)

[Immunohistochemistry Antibody Details	7](#_Toc218676628)

[Results	8](#_Toc218676629)

[[^18^F]ACI-12589 radiosynthesis for rat imaging	8](#_Toc218676630)

[[^18^F]Asyn-44 radiosynthesis for NHP imaging	10](#_Toc218676631)

[[^3^H]asyn-44 vs. [^3^H]ACI-12589 Autoradiography	12](#_Toc218676632)

[Metabolite study of [^18^F]asyn-44 in pig	13](#_Toc218676633)

[Metabolite study of [^18^F]asyn-44 in NHP	14](#_Toc218676634)

[Liver microsome study	15](#_Toc218676635)

[References	19](#_Toc218676636)

Materials and Methods

General

The asyn-44 precursor was provided by Sai Life Sciences Ltd. (Telangana, India). [^3^H]asyn-44 2.15 GBq/µmol, 37 MBq/mL) and [^3^H]ACI-12589 (3.03 GBq/µmol; 37 MBq/mL) were provided by Novandi Chemistry AB (Södertälje, Sweden). ACI-12589 reference standard and precursor were custom-synthesized by Novandi Chemistry AB (Södertälje, Sweden). All other chemicals and reagents were obtained from commercial vendors and were used as received without further purification.

Rats

Adult Sprague-Dawley rats were housed in conventional static caging on paper chip bedding. They were given nesting material, sunflower seeds and shelters for enrichment. The light cycle is 7 am on/7 pm off. They were allowed food and water ad libitum. Rodents were anesthetized using isofluorane in oxygen gas (5% at 2 L/min to induce unconsciousness and 1−2% at 1L/min to maintain) for tail-vein catheterization.

Pig

One female pig (crossbreed of Landrace × Yorkshire × Duroc) weighing 19 kg (approx. 9 weeks old) was used in the present study. The animal was sourced from a local farm and acclimatized for 5 days in an enriched environment prior to experiments. For PET scanning, anesthesia was induced approx. 8 h prior to scanning by intramuscular injection of 0.13 ml/kg Zoletil veterinary mixture (11.36 mg/mL xylazine, 11.36 mg/ml ketamine, 1.82 mg/ml butorphanol, 1.82 mg/ml methadone) and maintained by 12.5 mg/kg/h propofol infusion intravenous (i.v.). Femoral arteries and mammary veins were used for arterial blood sampling and i.v. access, respectively. Endotracheal intubation allowed for ventilation with 20 % oxygen in air at 10 ml/kg. Urine catheter was placed to avoid discomfort and stress. The animals were closely monitored throughout the experiment, with peripheral O_2_ and end-tidal CO_2_ saturation, heart rate, blood pressure, and temperature. After completion of the experiment, the animals were terminated by 15 ml pentobarbital i.v. injection.

NHP veterinary care, housing, and enrichment program

The male macaque was housed in a cage adjacent to another male macaque so that they could interact as much as possible without harmful physical contact. In the cage environment, species-specific behavior was encouraged by providing foraging feeding methods, perches, food puzzles, and various toys such as dental balls and paper for shredding. In addition, diverse food options, including a variety of fresh fruits, vegetables, and treats such as popcorn, peanuts, prima-treats and other items, provided different tastes, smells, colors, sizes, shapes and densities. Visual stimulation was provided by mirrors and a television played in the room during daylight hours. At various times a radio was played to provide additional auditory stimulation. Water was available ad libitum until the time of the PET scans. A full-time environmental enrichment specialist was assigned to the non-human primate floor of the animal facility. Enrichment techniques utilized by the enrichment specialist fell into four category types: treats, foraging, toys, and sensory/human. For example: 1) treats included popcorn, vegetables (carrots, turnips, sweet potato), dried fruit (pineapple, raisins, cranberries), and fresh fruits (grapes, apples, oranges); 2) foraging included puzzles filled with fruit; 3) toys stimulated visual and tactile senses and cognitive abilities, and included plastic toys of various shapes and approved destructible paper items; and 4) sensory/human enrichment included mirrors, television, and radio. Human interaction was provided at least 4 times per week through positive postures, gestures, and spoken interactions, as well as the placing of food treats in the cages. The daily record was monitored by animal caretakers, and the behaviors of all non-human primates were evaluated routinely for signs of psychological distress. Any non-human primate showing signs of psychological distress received special attention in terms of additional environment enrichments from the veterinary staff and the enrichment specialist.

[^18^F]Asyn-44 radiosynthesis for rat imaging (CAMH)

[^18^F]Fluoride was produced with a MC-17 cyclotron (Scanditronix, Sweden) from an oxygen-18 enriched water target. The work was carried out in accordance with the radiation protection guidelines and regulations from the Canadian Nuclear Safety Commission and internal safety policies and procedures. The synthesis was carried out on a TRACERlab FX2 N radiosynthesis module. [^18^F]Fluoride (5–20 GBq) was trapped on an anion exchange cartridge (HCO_3_; Synthra, Germany) and was eluted with a solution of 1.35 mg potassium bicarbonate and 14 mg Kryptofix® 222 in 0.9 mL methanol and 0.1 mL water. It was azeotropically dried at 85–110 °C under addition of 1 mL acetonitrile with nitrogen flow and vacuum. The reaction vessel was then cooled to 60 °C. The precursor (1 mg in 0.7 mL DMSO) was added, and the reaction mixture was stirred and heated at 100 °C. After 10 min, the reactor was cooled to 40 °C and the mixture was diluted with 4 mL mobile phase (acetonitrile/0.1 M ammonium formate 45:55). The product was purified by semipreparative HPLC (Luna C18 column (5 μm, 250×10 mm), acetonitrile/ 0.1 M ammonium formate 45:55, 6 mL/min) and the fraction eluting at ~25 min was collected in a flask containing 25 mL water and passed over a C18 solid-phase extraction cartridge. The product was eluted with 1 mL ethanol, followed by 9 mL saline. [^18^F]Asyn-44 was obtained in a total synthesis time of 1 h with a radiochemical purity (RCP) of >95%, a radiochemical yield (RCY) of 6 ± 2 % (decay corrected, *n* = 5) and a molar activity (A_m_) of 263 ± 121 GBq/μmol.

[^18^F]ACI-12589 radiosynthesis (CAMH)

[^18^F]Fluoride was produced with a MC-17 cyclotron (Scanditronix, Sweden) from an oxygen-18 enriched water target. Radiolabelling was performed on a TRACERlab FX2 N module according to a slightly modified literature procedure.^1^ Aqueous [^18^F]fluoride was obtained from the cyclotron and was trapped on an anion exchange cartridge (HCO_3_; Synthra, Germany). The [^18^F]fluoride was eluted with a solution of Bu_4_NH_2_PO_4_ (20 mM) in 50:50 acetonitrile/H_2_O (1 mL) and azeotropically dried at 85-110 °C under addition of acetonitrile (1 mL) with a flow of N_2_ and vacuum. A solution of mesylate precursor (2 mg) in anhydrous dimethyl sulfoxide (1 mL) was added and the reaction was stirred at 130 °C for 10 min. After dilution with water (3-4 mL), the reaction mixture was purified by semi-preparative HPLC (Luna C18(2) 10 μm 250×10 mm; acetonitrile/0.1 M ammonium formate 30:70). The product was collected in a flask containing water (25 mL) and passed over a C18 solid-phase extraction cartridge. After washing with water (10 mL), the product was eluted with ethanol (1 mL), followed by saline (9 mL). [^18^F]ACI-12589 was obtained in a RCP >95%, an activity yield of 8% (non-decay-corrected, *n* = 1) and a A_m_ = 37.4 GBq/µmol.

[^18^F]Asyn-44 radiosynthesis for pig imaging (Copenhagen University Hospital)

[^18^F]Fluoride was produced via the (p,n)-reaction in a cyclotron (CTI Siemens and Scanditronix, Rigshospitalet, Denmark) by irradiating [^18^O]H_2_O with a 11 MeV proton beam. The synthesis was carried out on a Scansys Laboratorieteknik synthesis module. [^18^F]Fluoride (10 GBq) was trapped on an anion exchange cartridge (HCO_3_; Synthra, Germany) and was eluted with a solution of 1.35 mg potassium bicarbonate and 14 mg Kryptofix® 222 in 0.9 mL MeOH and 0.1 mL water. It was azeotropically dried at 100 °C under addition of 1 mL acetonitrile with a N_2_ flow and vacuum. The precursor (1 mg in 0.7 mL dimethyl sulfoxide) was added, and the reaction mixture was heated at 100 °C. After 10 min, the reactor was cooled to 40 °C and the mixture was diluted with 4 mL mobile phase (acetonitrile/0.1 M ammonium formate 45 : 55). The product was purified by semi-preparative HPLC (Luna C18 column (5 μm, 250×10 mm), acetonitrile/0.1 M ammonium formate 50:50, 6 mL/min) and the fraction eluting at ~25 min was collected in a flask containing 25 mL water and passed over a C18 solid-phase extraction cartridge. The product was eluted with 1 mL ethanol, followed by 9 mL saline. [^18^F]Asyn-44was obtained in a total synthesis time of 1 h with a RCP of >95 %, a RCY of 5% (decay-corrected, *n* = 1) and a A_m_ = 28.44 GBq/µmol.

[^18^F]Asyn-44 radiosynthesis for NHP imaging (University of Pittsburgh)

Radiolabeling was performed manually in a lead-shielded hotcell using the manipulator arms. No-carrier added [^18^F]F^-^ was produced via the ^18^O(p,n)^18^F reaction (Siemens Eclipse HP cyclotron), transferred in [^18^O]H_2_O to the hotcell, and trapped (35.3 GBq (954 mCi)) on a Waters Sep-Pak Plus Light QMA (WAT023525) (previously conditioned with Et_4_NHCO_3(aq)_ solution (8 mL, 7 mg/mL), Milli-Q H_2_O (10 mL), then air (25 mL)). The QMA was eluted with Et_4_NHCO_3(aq)_ solution (1 mL, 7 mg/mL), then CH_3_CN (1 mL), then air (1 mL) (0.3 GBq (8.7 mCi) remained on the QMA), and collected in a 5-mL V-vial. The solvents were evaporated under argon flow at ~110 ^o^C, then the Et_4_N^18^F was azeotropically dried with CH_3_CN aliquots (2 mL, then 1 mL) at ~110 ^o^C under argon flow to give a white film on the V-vial wall (23.5 GBq (634 mCi)). Radiolabeling precursor (1.1 mg) was dissolved in DMF (0.5 mL), added to the V-vial, the V-vial was capped, heated at ~110 ^o^C for 15 min, then cooled. The solution was diluted with HPLC solvent (0.5 mL, 45:55 v/v CH_3_CN/0.1 M NH_4_HCO_2_ pH 4.2), loaded onto the HPLC loop, the V-vial was rinsed with HPLC solvent (0.5 mL), the solvent was loaded onto the HPLC loop (1.2 GBq (32.1 mCi) remained in the V-vial), and the sample was purified by preparatory-HPLC (Phenomenex Luna, 5 µm, C18(2), 100 Å, 250 x 10 mm + guard; 4 mL/min, 254 nm). The desired HPLC fraction was collected in Milli-Q H_2_O (50 mL), passed through a Waters C_18_ Sep-Pak (WAT020515) (previously conditioned with EtOH (10 mL) then Milli-Q H_2_O (10 mL)), and the Sep-Pak was rinsed with Milli-Q H_2_O (10 mL) (0.8 GBq (22.7 mCi) trapped on the Sep-Pak). The radiotracer was eluted from the Sep-Pak with EtOH (1 mL), then 0.9% sterile saline (14 mL), passed through a 0.2 micrometer filter, and collected in a sterile vial to afford [^18^F]asyn-44 (0.7 GBq (18.0 mCi)); 1.9% radiochemical yield (not decay-corrected); 99.8% radiochemical purity (Phenomenex Gemini, 5 μm, NX-C18, 110 Å, 100 x 4.6 mm; 35:65 v/v CH_3_CN/0.1 M NH_4_HCO_2_ pH 4.2; 1 mL/min; 254 nm); apparent A_m_ = 24.9 MBq/nmol (0.67 mCi/nmol); pH 5). Radiochemical identity was confirmed by co-injection with asyn-44 standard. The endotoxin level of the final formulation was <2.00 EU/mL (Charles Rivers Endosafe).

Radiometabolite Identification

**Chemicals and Materials**

All reagents were purchased from Sigma-Aldrich Merck (Milan, Italy) unless otherwise stated. LC-MS grade acetonitrile and formic acid were obtained from Honeywell (Charlotte, NC, USA), ultrapure water for LC-HRMS analyses was generated using a Direct-Q 3UV water purification system (Merck Millipore, Milan, Italy). Pooled rat liver microsomes (Sprague Dawley strain), derived from 25 donors and supplied as a 0.5 mL aliquot with a protein concentration of 20 mg/mL, were acquired from Thermo Fisher Scientific, Gibco (Waltham, MA, USA) and stored at –70 °C until use. Nicotinamide adenine dinucleotide phosphate (NADPH), employed as the phase I metabolic cofactor, was purchased from BLD Pharmatech GmbH (Reinbek, Germany).

***In silico* metabolite prediction**

The metabolites of Asyn-44 were predicted using “phase I metabolism” in GLORYx, a web-based machine learning model for simulating metabolic reactions.^2^ The metabolites with the major highest probability score (shown in **Supplemental Table 4**) were then incorporated in an inclusion list for LC-HRMS/MS analysis.

**Liquid Chromatography High Resolution Mass Spectrometry (LC-HRMS) analysis**

Metabolite analysis was performed using a LC-HRMS system from Thermo Fisher Scientific (Waltham, MA, USA), which included Ultimate 3000 ultra-high performance liquid chromatograph (UHPLC) coupled to a Q-Exactive quadrupole-Orbitrap mass spectrometer equipped with a heated electrospray ionization (HESI) source. The chromatographic module featured a binary solvent pump, vacuum degasser, temperature-regulated autosampler (maintained at 7 °C), and a column oven set to 25 °C. Mass spectrometric detection was carried out in positive ionization mode. For chromatographic separation, a Poroshell 120 EC-C18 column (100 × 3.0 mm I.D., 2.7 µm) protected by a matching guard cartridge (5 × 3 mm I.D., 2.7 µm) (Agilent Technologies, Milan, Italy) was used. The mobile phase consisted of water and acetonitrile, each containing 0.1% formic acid. The elution program included a linear gradient from 5% to 95% acetonitrile between 2.0 and 20.0 min, followed by an isocratic hold at 95% for 5 min. The mobile phase was then returned to 5% acetonitrile at 25.1 min and held for re-equilibration until 30.0 min. The Orbitrap analyzer was operated in full scan mode across a mass range of m/z 50–750. For structural elucidation, data-dependent acquisition was conducted on pooled quality control (QC) samples to generate MS/MS spectra for both the parent compound and its major metabolites. Ionization source parameters were configured as follows: capillary temperature, 320 °C; vaporizer temperature, 300 °C; spray voltage, 3.8 kV; sheath gas flow rate, 55 arbitrary units (au); auxiliary gas, 30 au; and S-lens RF level set at 55 au. The instrument was calibrated prior to analysis, and a lock mass list was applied to ensure high mass accuracy. Data were acquired using Xcalibur 3.0 software (Thermo Fisher Scientific, San Jose, CA, USA), with both full-scan and data-dependent MS/MS modes enabled throughout a 1-30 minute acquisition window. In full scan, the automatic gain control target was set at 3 × 10⁶ with a resolution of 140,000 (FWHM) and a maximum injection time of 500 ms. The mass range spanned from m/z 50 to 750. In data-dependent acquisition mode, MS/MS spectra were collected with an automatic gain control target of 5 × 10⁵, a resolution of 17,500, and a maximum injection time of 100 ms. Fragmentation was performed using a normalized collision energy of 20 au, with an isolation window of *m/z* 0.4, a loop count of 2, and a dynamic exclusion duration of 5.0 seconds.

**Quantitation of asyn-44 in microsomal samples**

100 μM working solutions of asyn-44 was used to prepared five non-zero calibration points and build the calibration curve 0.01–1 µM (0.01, 0.05, 0.1, 0.5, and 1 µM). All dilutions were prepared in 0.1 M potassium phosphate buffer (pH 7.4) and 50 μL of ice-cold acetonitrile containing internal standard. Linear regression was calculated on three replicates for each calibration point. Peak areas of asyn-44 divided for internal standard peak areas were plotted against nominal concentrations and the linearity was assessed by the coefficient of determination (*R^2^*) with the method of the least squares, which was greater than 0.99 for all analytes, and mean precision (CV%) obtained from the back-calculated concentration within ± 15 % of the nominal concentrations for all calibration points and within ± 20 % of the lower limit of quantification (0.01 µM), while the limit of detection was 0.003 µM.

**Identification of asyn-44 metabolites**

Metabolite profiling of asyn-44 was performed using Compound Discoverer 3.3 (Thermo Fisher Scientific, Waltham, MA, USA) with the built-in *MetID* workflow. The analysis included retention time alignment, detection of expected Phase I metabolites, isotopic pattern confirmation, and grouping across samples. Structural annotation was aided by FISh (Fragment Ion Search) scoring, which matched experimental MS/MS spectra to predicted fragments. The Compound Class Scoring tool was also used to flag unknown compounds that share common fragments to cluster metabolites with parent compound.

**Estimating the intrinsic clearance (*CL_int_*) of Asyn-44**

The rate of depletion (*rd*) was calculated as ***(pmol/min/mg) =*** ***(*Δ*C× 1000)/*** ***(B ×T)*** where Δ*C*=[concentration at 0 min]– [concentration at 30 min (μM)]; *B* is the microsome protein concentration (mg/ml); *T* is the incubation time (min); and 1000 is the conversion factor from nmol to *p*mol.^3^ Intrinsic clearance (*Cl_int_*) of asyn-44 was determined by normalizing the mean peak area ratios from duplicate measurements at each time point to time zero. The decay profile was fitted with a one-phase exponential decay model (GraphPad Prism 8.0.2) to obtain the elimination rate constant (*k*) using ***C_t_ = C_0_e^−kt^*** where *t* = 30 for 30-min incubation and *C* is the concentration of the sample at the corresponding incubation time. The *in vitro* half-life (*t₁_/_₂*) was calculated as ***t_1/2_ (min) = ln (2)/k***.^4^ Subsequently, *in vitro* intrinsic clearance (*Cl_int, in vitro_*) was calculated as ***Cl_int, in vitro_ (µl/min/mg) = V × 0.693/ t_1/2_*** where *V* is the incubation volume per mg of microsomal protein.^3^ The *in vivo* intrinsic clearance (*Cl_int, in vivo_*) was extrapolated by scaling *in vitro* values with microsomal protein content (61 mg/g liver) and liver weight (40 g/kg body weight) for rat.

Brain Tissue Patient Demographics

**Supplemental Table 1 Patient demographics of brain tissue used for ARG and IHC.**

| **Identifier** | **Diagnosis** | **Age at Death**  **(Years)** | **Gender**  **(M/F)** | **Brain region** | **Pathology** | **Tissue Preparation** |
| --- | --- | --- | --- | --- | --- | --- |
| MSA1 | MSA (cerebellar) | 68 | M | Dentate nucleus | α-syn (3+), tau (0), Aβ (0), TDP-43 (0) | FFPE |
| MSA2 | MSA (cerebellar) | 54 | M | Cerebellum | α-syn (+), Aβ (-), Tau (-) | Fresh Frozen |
| PD1 | PD (non demented) | 78 | M | Cingulate gyrus | α-syn (2+), tau (0), Aβ (0), TDP-43 (0) | FFPE |
| HC1 | Healthy control | 66 | F | Cerebellum | α-syn (-), Aβ (-), Tau (-) | Fresh Frozen |
| AD1 | Alzheimer’s disease | 70 | M | Frontal Cortex | α-syn (-), Aβ (+), Tau (+) | Fresh Frozen |
| LBD1 | Lewy body dementia | 75 | M | Putamen | α-syn (+), A2B1C0, CAA, LATE | Fresh Frozen |

Immunohistochemistry Antibody Details

**Supplemental Table 2*:* Primary antibodies selected for immunohistochemistry.**

| **Primary Antibodies** | **Source/Cat No** | **Host and clonality** | **Antibody dilution** | **Incubation** |
| --- | --- | --- | --- | --- |
| anti-alpha synuclein  (phosphorylated S129) | Abcam/ ab184674 | Mouse Monoclonal | 1:500 | O/N at 4ºC |
| Anti Phosphorylated α-Synuclein | FUJIFILM Wako / 015-25191 | Mouse Monoclonal | 1:5000 | O/N at 4ºC |
| Purified anti-β-Amyloid, 17-24 Antibody | BioLegend / 800701 | Mouse Monoclonal | 1:6000 | O/N at 4ºC |

**Supplemental Table 3 Secondary antibodies selected for IHC.**

| **Secondary Antibodies** | **Source/Cat No** | **Host and clonality** | **Antibody dilution** | **Incubation** |
| --- | --- | --- | --- | --- |
| Goat Anti-Mouse IgG H&L | Abcam/ab205719 | Goat polyclonal | 1:10000 | 1h at RT |
| Agilent Dako EnVision+ Single Reagent (HRP. Mouse) | Agilent Technologies /  K400111-2 | Goat peroxidase labeled polymer | Undiluted | 30min at RT |

Results

[^18^F]ACI-12589 radiosynthesis for rat imaging

**Supplemental Figure 1 Preparative HPLC chromatogram of the [^18^F]ACI-12589 synthesis.**

**Supplemental Figure 2** **Analytical HPLC chromatogram of the [^18^F]ACI-12589 final product for rat imaging with 99% RCP and apparent A_m_ = 37.4 GBq/µmol (1.01 Ci/µmol).**

**Supplemental Figure 3 Analytical HPLC chromatogram of co-injection of [^18^F]ACI-12589 and standard.**

[^18^F]Asyn-44 radiosynthesis for NHP imaging

**Supplemental Figure 4** **Analytical HPLC chromatogram of the asyn-44 precursor (92.6% purity).**

**Supplemental Figure 5** **Analytical HPLC chromatogram of the [^18^F]asyn-44 final product for NHP imaging with 99.8% radiochemical purity and apparent A_m_ = 24.9 GBq/µmol (0.67 Ci/µmol).**

**Supplemental Figure 6** **Analytical HPLC chromatogram of co-injection of [^18^F]asyn-44 and asyn-44 standard.**

[^3^H]asyn-44 vs. [^3^H]ACI-12589 Autoradiography

**Supplemental Figure 7.** **Semi-quantitation of [^3^H]asyn44 and [^3^H]ACI-12589 heterologous blocking by autoradiography in FFPE postmortem PD1 and MSA1 cases.** (**A**) Total binding of [^3^H]asyn44 (20 nM), non-specific binding in the presence of 10 µM of unlabeled ACI-12589 and specific binding expressed as counts/min/mm^2^ in MSA1 and PD1 brains. (**B**) Total binding of [^3^H]ACI-12589 (20 nM), non-specific binding in the presence of 500 nM of unlabeled asyn-44 and specific binding expressed as counts/min/mm^2^ in MSA1 and PD1 brains. (**C**) Percent displacement of [^3^H]asyn44 by 500 nM unlabeled asyn-44 and 10 µM unlabeled ACI-12589 in MSA1 and PD1. (**D**) Percent displacement of [^3^H]ACI-12589 by 10 µM unlabeled ACI-12589 and 500 nM unlabeled asyn-44 in MSA1 and PD1.

Metabolite study of [^18^F]asyn-44 in pig

**Supplemental Figure 8** **[^18^F]Asyn-44 Metabolite Profile in Pig.** Chromatogram showing the parent compound peak at 2.5, 10, 30, 70 and 120 min and no formation of lipophilic metabolites.

Metabolite study of [^18^F]asyn-44 in NHP

**Supplemental Figure 9 Metabolite analysis of [^18^F]asyn-44 in NHP.** HPLC chromatograms of venous blood samples at 2, 10, 30, 45, 60 and 90 minutes; t_R_ ~3 min polar metabolite, t_R_ ~10 min parent compound, t_R_ ~16 min lipophilic metabolite.

Liver microsome study

Using a gradient-based chromatographic method with ACN and water containing 0.1% formic acid as the mobile phases and a C_18_ core-shell stationary phase column, Asyn-44 eluted at a *R_T_* of 8.49 min (Supplemental Figure 10A). In positive electrospray ionization mode (ESI+), it was detected as the protonated molecular ion [M+H]^+^ at *m/z* 377.1224. MS/MS analysis of the precursor ion produced a characteristic fragmentation pattern (Supplemental Figure 10B), with proposed structures for the major fragments annotated in the spectrum. Chromatograms from liver microsomal incubations at 0 and 30 min were processed using Compound Discoverer software with the *MetID* workflow.

**Supplemental Figure 10 Asyn-44 Mass Spectrometry.** (**A**) MS1 of the Asyn-44 molecular ion and (**B**) its MS/MS fragmentation spectrum.

**Liver Microsome Stability**

The chemical stability of the test compound was confirmed by comparing its concentration at time zero (T₀) with that in the negative control (incubation without NADPH). The concentration of the parent compound in the negative control was 118.9 % relative to T₀, indicating no significant degradation in the absence of metabolic cofactors. This suggests that the compound is chemically stable under the present assay conditions. The rate of depletion was used to assess the extent of metabolism by measuring substrate depletion over the 30-min incubation. The mean rate of depletion was found to be 252.47 ± 6.34 *p*mol/min/mg. Intrinsic clearance *in vitro* (*Cl_int in vitro_;* µl/min/mg) was determined to be 34.95 ± 2.98, the elimination rate constant (*k*) resulted 0.0699 ± 0.00595 min⁻¹ and the half-life (t_1/2_) = 9.95 ± 0.85 min. Consequently, its intrinsic clearance *in vivo* (*Cl_int in vivo_;* mL/min/Kg) was estimated to be 85.27 ± 7.26. Testosterone was used as an experimental control and was fully metabolized within 30 min. In comparison to testosterone (with an experimental *Cl*_int in vitro_ = 53 µL/min/mg), asyn-44 exhibited a slight slower metabolism.

***In silico* metabolite prediction**

The metabolites of asyn-44 predicted using GLORYx, a web-based machine learning model for simulating metabolic reaction are shown in Supplemental Table 4 in order of the probability score provided by the software. The results indicate that all the metabolites proposed are products of oxidation. Specifically, oxidated metabolites at the N-pyrrolidine ring have the highest priority score (3.07 and 2.90), while oxidated metabolite at sulphur has a lower priority score (2.69). Less probable metabolites were also predicted by the software.

Supplemental Table 4 Predicted top metabolite structures of asyn-44 by GloryX with priority score.

| *N°* | *Structure* | *Priority score* |
| --- | --- | --- |
| M1 |  | 3.07 |
| M2 |  |  |
| M3 |  | 2.90 |
| M4 |  |  |
| M5 |  | 2.69 |
| M6 |  | 1.06 |
| M7 |  | 1.02 |
| M8 |  | 1.01 |
| M9 |  | 0.87 |
| M10 |  | 0.71 |

In addition to the major oxidation species (M1), a minor oxidated species (M2; Supplemental Figure 11D) was detected with the same protonated molecular ion [M+H]^+^ as M1 at *m/z* 393.1172, eluting at *R_T_* of 8.75 minutes (Supplemental Figure 11E). The MS/MS analysis of the precursor ion produced a fragmentation pattern reported in Supplemental Figure 11F with the major fragments annotated in the spectrum. The proposed structure for this metabolite may correspond to one of the oxidative metabolites proposed (Supplemental Figure 11D). Supplemental Figure 11F illustrates the fragmentation pattern, with the proposed structures of the major fragments—assigned by Compound Discoverer—annotated within the spectrum. A third metabolite (M3; Supplemental Figure 11G) eluted at a retention time (*R_T_*) of 15.19 minutes (Supplemental Figure 11H), with a protonated molecular ion [M+H]+ at *m/z* 355.1002, consistent with the parent compound minus three hydrogen atoms and one fluorine atom. The MS/MS fragmentation spectrum of the molecular ion did not yield any significant fragment ions (Supplemental Figure 11I). The proposed structure, shown in Supplemental Figure 11G, suggests the formation of a double bond within the pyrrolidine ring and the loss of the fluorine atom, followed by aromatization. Several minor metabolites, all resulting from oxidation of the parent compound, were also detected.

Figure 11 Proposed structures (A, D, G), LC-HRMS extracted ion chromatograms (B, E , H), and MS/MS spectra (C, F, I) of metabolites M1, M2, and M3, formed in rat liver microsomes under phase I metabolic conditions.

References

1.	Smith R, Capotosti F, Schain M, et al. The α-synuclein PET tracer [18F] ACI-12589 distinguishes multiple system atrophy from other neurodegenerative diseases. *Nat Commun*. 2023;14(1):6750. doi:10.1038/s41467-023-42305-3

2.	de Bruyn Kops C, Šícho M, Mazzolari A, Kirchmair J. GLORYx: Prediction of the Metabolites Resulting from Phase 1 and Phase 2 Biotransformations of Xenobiotics. *Chem Res Toxicol*. 2021;34(2):286-299. doi:10.1021/acs.chemrestox.0c00224

3.	Knights KM, Stresser DM, Miners JO, Crespi CL. In Vitro Drug Metabolism Using Liver Microsomes. *Curr Protoc Pharmacol*. 2016;74(1):7.8.1-7.8.24. doi:https://doi.org/10.1002/cpph.9

4.	Tang LWT, Chan ECY. Quantification of the irreversible fibroblast growth factor receptor inhibitor futibatinib by UPLC-MS/MS: Application to the metabolic stability assay in human liver microsomes for the estimation of its in vitro hepatic intrinsic clearance. *J Pharm Biomed Anal*. 2022;214:114731. doi:https://doi.org/10.1016/j.jpba.2022.114731
